# Nutritional Properties of Rice Varieties Commonly Consumed in Italy and Applicability in Gluten Free Diet

**DOI:** 10.3390/foods10061375

**Published:** 2021-06-14

**Authors:** Giorgia Vici, Diego Romano Perinelli, Dalia Camilletti, Flora Carotenuto, Luca Belli, Valeria Polzonetti

**Affiliations:** 1 School of Biosciences and Veterinary Medicine, University of Camerino, Via Gentile III da Varano, 62032 Camerino, MC, Italy; dalia.camilletti@unicam.it (D.C.); flora.carotenuto@studenti.unicam.it (F.C.); luca.belli@unicam.it (L.B.); valeria.polzonetti@unicam.it (V.P.); 2 School of Pharmacy, University of Camerino, Via Gentile III da Varano, 62032 Camerino, MC, Italy; diego.perinelli@unicam.it

**Keywords:** rice, amylose content, glycaemic response, gluten-free diet

## Abstract

Gluten-free diets are often characterized by an inadequate intake of nutrients and are generally monotonous for the limited number of products celiac patients can use. As rice is the most used cereal by celiac consumers, studying rice varieties nutritional characteristics is of interest to manage diet quality and variety. Proteins, total carbohydrates and amylose content of six rice varieties (Ribe, Vialone Nano, Carnaroli, Arborio, Basmati, and Fragrance) were analyzed. Analyses were performed in raw products and after boiling, stewing, and microwaving. A decrease of proteins and total carbohydrates amount was observed in cooked rice. The same was reported for amylose content with boiling showing the highest loss (average retained amylose 53%). Considering amylose percentage with respect to total carbohydrates, each variety showed either an increase or a decrease depending on cooking method. The highest values were obtained with stewing above all for Basmati rice and Arborio rice. However, exceptions can be underlined as Carnaroli rice, showing the highest percentage when boiled. In this context, nutritional characteristics of cooked rice varieties appear to be of great importance to increase specific nutritional knowledge to better manage gluten-free diets.

## 1. Introduction

Celiac disease is a T cell mediated intestinal disorder, genetically inherited, which affects the small intestine by damaging intestinal villi and consequently compromising nutrient absorption [1]. The trigger responsible for an abnormal immune response in genetically susceptible individuals is wheat protein gluten [2]. The only effective treatment for celiac disease patients is to follow a life-long gluten-free diet [3]. A gluten-free diet is necessary for celiac patients, but such a specific diet could be complicated by the effort involved in selecting high quality gluten-free foods and in maintaining a balanced and varied dietary regimen [4]. The use of commercial gluten-free foods, often rich in fats and hypercaloric, can contribute to the risk of overweight and obesity [5]. Moreover, according to Agarwal et al., the risk of developing metabolic syndrome and fatty liver disease in celiac patients following a gluten-free diet is high, especially with the consumption of commercially available gluten-free products [6]. As a gluten-free diet is followed by celiac patients for their entire life, it is essential to guarantee nutrient balance and to avoid deficiencies in order to maintain a healthy status [7,8].

Indeed, gluten-free diets are often characterized by an inadequate intake of nutrients, such as excess fats and sugar, and a lack of vitamins (i.e., vitamin B12, folate, vitamin C) and minerals (i.e., calcium, selenium, zinc and magnesium) [9].

Therefore, to avoid nutrient deficiency, it is fundamental to follow a healthy balanced diet [10]. Mediterranean diet (MD) has been widely described to positively impact health status [11]. According to the MD dietary guidelines, it is necessary to consume different food categories every day to guarantee nutrient balance in the diet [12,13]. Cereals are one of the pillars of the Mediterranean diet and they should be part of every daily meal [14,15]. In this perspective, it is fundamental to vary among different types of cereals available and for each type, changing current varieties. This aspect is fundamental to follow a balanced diet, especially for celiac consumers who have a limited number of products to select. Among cereals, rice is the mostly used by celiac consumers [16,17]. Consequently, it can be useful to better exploit nutritional characteristics of rice varieties. In general, cereals contain carbohydrates, proteins, vitamins, minerals, antioxidants and phytochemicals [18,19]. However, attention should be paid to carbohydrate contents of different cereals and varieties and, in particular, to amylose, one of the components of starch. Amylose has a slightly branched structure [20] while amylopectin, the other starch component, has a highly branched structure [21]. Amylose is less available for degradation by digestive enzymes because of the reduced surface area per molecule than amylopectin, thus inducing a lower impact on glycaemic response [22,23,24]. At the same time, it is evident that high amylose starches are less available for digestion than low amylose starches [22]. Moreover, amylose is converted into resistant starch, which is involved in improving the parameters of glucose and insulin homeostasis and has an hypocholesterolaemic effect [25,26].

Beyond amylose content, amylopectin content plays a role in starch digestion rate. For its highly branched structure, amylopectin is more available for degradation than amylose [22,23,24]. Anyway, the degree of branching and the length of chains of amylopectin molecule must be considered as an important parameter in starch digestion [27,28].

Although a high amylose content ameliorates the nutritional characteristics of rice, it is essential to consider that it influences rice taste and consequently consumer acceptance [29]. Indeed, different studies highlight that a low amylose concentration improves the texture and the eating quality of rice [30,31,32].

It can be gathered that a high amylose concentration negatively impacts on rice taste but in parallel exerts nutritional benefits. Accordingly, selecting a high amylose rice results the preferable choice for health [33]. Moreover, it must be considered that cereals, including rice, constitute a part of a balanced plate in which other foods, such as fruits and vegetables, plant-based fats, healthy proteins, and nuts, contribute to improve the quality of the meal [34].

Furthermore, it is widely known that the quality of the diet and nutrient balance depend also on the cooking methods used. Indeed, cooking methods differently affect the nutritional profile of foods [35,36,37,38]. The knowledge of cooking methods impact on nutritional quality of cereals could guide nutritionist or expert in nutrition in the choice of the appropriate cooking method to better manage glucose response, thus improving the general quality of the diet [39].

Consequently, the study of the nutritional characteristics of cereals could be of great interest for selecting them according to specific and personalized nutritional needs. In the case of celiac patients, this knowledge can contribute to better manage the quality of the diet, to make it more varied and balanced [40].

This study aims to analyse protein and carbohydrates content, in particular amylose content, of different varieties of rice commonly consumed in Italy in order to ameliorate the quality of gluten-free diets, to promote healthy dietary habits, but also to guide food choices for specific nutritional requirements (i.e., daily diet, physical exercise) in celiac patients.

## 2. Materials and Methods

### 2.1. Materials

Six varieties of rice were purchased from an Italian supermarket: Ribe (Contiriso Riseria Conti Francesco S.R.L; Camerino, Italy), Vialone Nano (Selex S.p.a; Camerino, Italy), Carnaroli (Selex S.p.a., Camerino, Italy); Arborio (Selex S.p.a.; Camerino, Italy); Basmati (Selex S.p.a; Camerino, Italy), Fragrance (Selex S.p.a.; Camerino, Italy) (Table 1). Sulphuric acid (analytical grade), D (+) -glucose anhydrous, iodine and potassium iodide were purchased from J.T. Baker (Holland). Anthrone analytical standard (purity > 97%), amylose (from potato) and Bovine Serum Albumin (BSA) were purchased from Sigma-Aldrich (USA). Bradford reagent was from Bio-Rad Laboratories, Inc., Italy.

### 2.2. Sample Preparation

For raw rice samples (uncooked), rice grains were ground into a fine flour using a mechanical grinder (Robert Bosch Engineering Company, Germany) before analysis.

Rice was also processed using three cooking methods: boiling, stewing and microwaving.

For boiling: 50 g of rice were immersed in 500 mL of water preheated at 100 °C and boiled for the time indicated on product’s label (Table 2). Then, the cooking water was removed, and cooked rice was collected in a filter paper.

For stewing: 50 g of rice was placed in a pot and toasted for 30 s. Then, aliquots of water were added every 3 min for the cooking time indicated on product’s label (Table 2). The water utilized for this cooking method depends on different ability to absorb water.

For microwaving: 50 g of rice were placed in a suitable container and cooked in a microwave oven at a power of 750 W using 166 mL of water for the time indicated in Table 2.

The cooked materials from the three methods were dried in an oven at 37 °C until complete drying and then ground into a fine flour using a mechanical grinder (Robert Bosch Engineering Company, Germany) before analysis.

### 2.3. Protein Content Determination (Bradford Assay)

Total protein content in rice samples was determined by the Bradford assay. For the analysis, 0.010 g of the samples were weighted out and 10 mL of NaOH 0.1 M was added and heated at 50 °C for 20 min. The analysis was performed using 60 µL of the sample, Coomassie Blue as reagent and bovine serum albumin (BSA) for the calibration curve (y = 0.0060 x + 0.0169; R^2^ = 0.993). The analysis was carried out according to the reported procedure by Bradford [41] and protein content was quantified spectrophotometrically at 595 nm. Analyses were performed in triplicates.

### 2.4. Total Carbohydrate Determinaton (Anthrone Assay)

Anthrone assay is a spectrophotometrical method for the determination of the total carbohydrates content. For the test, 0.010 g of the sample were dissolved in NaOH 0.1 M at 50 °C for 20 min and diluted up to 100 mL with ultrapure water. Then, 2 mL of the anthrone reagent solution (0.040 g of anthrone in 20 mL of concentrated sulfuric acid) were added to 1 mL of sample diluted solution and boiled for 10 min in a water bath. After cooling down at room temperature, the absorbance of solution was measured spectrophotometrically at 630 nm. The total carbohydrate content of the samples was determined by building up a calibration curve using pure glucose standard solutions (y = 0.0140 + 0.0267; R^2^ = 0.990). Analyses were performed in triplicates.

### 2.5. Amylose Content Determination (Iodine Assay)

Iodine colorimetric method was used to determine the amylose content. For the test, 0.010 g of the sample were dissolved in NaOH 0.1 M at 50 °C then neutralized with HCl 4 M and dilute up to 50 mL with ultrapure water. The iodine reagent solution was diluted in ultrapure water of a stock solution containing 7% *w*/*w* iodine and 5% *w*/*w* potassium iodide in a hydro-alcoholic mixture (90% *v*/*v* of ethanol). Then, 0.5 mL of the iodine reagent solution (0.2% *w*/*w* of iodine in water) were added to 10 mL of sample diluted solution. After mixing, the absorbance of solution was measured spectrophotometrically at 625 nm. The amylose content of the samples was determined by building up a calibration curve using pure amylose standard solutions (y = 0.1828 + 0.1320; R^2^ = 0.994). Analyses were performed in triplicates.

### 2.6. Statistical Analysis

Data obtained were recorded in GraphPad Prism version 9.1.0 for Windows, (GraphPad Software, San Diego, CA, USA) and Two-way ANOVA followed by Tukey’s multiple comparisons test was performed taking into account rice varieties and cooking methods. Each *p* value was adjusted to account for multiple comparison with family-wise alpha threshold and confidence levels set at 0.05 (95% confidence interval).

## 3. Results

### 3.1. Protein Content Determination

Protein amounts detected in raw rice were comparable with ones reported on the nutritional labels (Table 1) and range from 5.89 to 8.71 g per 100 g of product among the different varieties (Table 3). Protein contents of analyzed raw rice were statistically significantly different each other. All selected cooking methods reduced detectable proteins amount in all rice in comparison with the corresponding raw rice (Table 3).

In general, boiling causes the highest loss of detectable protein content with an average amount of retained proteins of 79%, with Basmati rice showing the lowest percentage (63.67 ± 0.40%) and Arborio rice showing the highest (87.70 ± 0.38%).

An average of 88% of retained proteins was observed after stewing. Also in this case, Basmati rice showed the lowest percentage (79.01 ± 0.45%) but Vialone Nano rice showed the highest (96.10 ± 0.30%). After the microwaving cooking process, the average of detectable proteins (around 89%) was similar to the one detectable after stewing process. In this case, the highest value was associated to Ribe rice (97.89 ± 0.21%) while the lowest to Fragrance rice (81.40 ± 0.41%).

Comparing different cooking methods process impact on detectable proteins amount, differences were observed among all the analyzed rice underlining the influence of both cooking methods and rice varieties on proteins amounts. Multiple comparisons are reported on Table S1. 

### 3.2. Total Carbohydrates

Carbohydrates amounts in raw products were comparable to those reported on product’s labels for all analyzed rice (Table 1) ranging from 75.50 to 77.06 g/100 g of rice (Table 4).

No differences were observed comparing raw rice varieties. However, a decrease in carbohydrates content occurred after each cooking process.

Boiling process caused the highest decrease in total carbohydrates with an observed average of remained carbohydrates of 53%. In this case, Vialone Nano rice showed the lowest percentage (47.64 ± 0.30%) and Basmati rice showed the highest (64.05 ± 0.75%).

Stewing led to an average of 70% of retained carbohydrates after treatment. Also in this case, Vialone Nano rice showed the lowest percentage (55.85 ± 0.56%) while Carnaroli rice showed the highest (89.45 ± 0.30%). Microwaving was the cooking methods that preserved the highest percentage of carbohydrates, average of 90%. Vialone Nano rice showed the lowest percentage (83.72 ± 0.48%) while Carnaroli rice the highest (93.51 ± 0.38%) (Table 4).

Comparing the impact of the different cooking methods on detectable total carbohydrates amount, differences were observed among all the analyzed rice with only few exceptions regarding stewed Ribe rice compared with boiled Basmati rice, stewed Ribe rice compared to stewed Carnaroli, stewed Carnaroli rice compared with microwaved Fragrance rice microwaved, and stewed Vialone Nano rice compared with boiled Arborio rice. Multiple comparisons are reported on Table S2.

### 3.3. Amylose Content

Raw amylose content ranges between 24.36 and 32.64 g/100 g. Among rice varieties, amylose content of raw products was statistically different with the exception of Fragrance rice compared to Arborio rice (adjusted *p* value = 0.9993) and Carnaroli rice compared to Vialone Nano rice (adjusted *p* value > 0.9999).

The loss of amylose after cooking followed a similar trend as observed for total carbohydrate with boiling process showing the highest loss followed by stewing and then microwaving (Table 5).

The average percentage of remained amylose after boiling resulted around 53% with the highest value obtained in case of Carnaroli rice (63.07 ± 2.17%) while the lowest in case of Arborio rice (42.01 ± 0.56%). Stewing caused an average amylose remaining of 76% with Ribe rice showing the lowest value (68.76 ± 0.69) and Fragrance rice the highest (85.25 ± 1.28%). Microwaving led to the highest average amylose remaining (average of 88%) with the lowest value obtaining for Basmati rice (79.23 ± 0.08%) and the highest with Fragrance rice (92.97 ± 0.92).

Moreover, in the case of amylose content, significant differences were found comparing different rice and different cooking methods with the exception of microwaved Ribe rice compared to stewed Basmati. Multiple comparisons are reported on Table S3.

### 3.4. Amylose Percentage with Respect to Total Carbohydrates

Amylose percentage with respect to total carbohydrates was calculated both in raw and in cooked rice (Table 6).

Percentage of amylose with respect to total carbohydrates ranges from 31.61% to 42.38% in raw products. Statistically significant differences were found in raw products depending on rice varieties with few exceptions regarding Ribe rice compared with Fragrance rice and Arborio rice, Carnaroli rice compared with Vialone Nano rice, and Fragrace rice compared with Arborio rice.

After cooking process application, differences were found in terms of percentage of amylose with respect to total carbohydrates depending on both rice variety and cooking methods.

In particular, a significant decrease in the percentage of amylose with respect to total carbohydrates was observed after boiling in case of Basmati rice, Fragrance rice and Arborio rice while no statistical changes were observed in case of Ribe rice. On the other hand, a statistically significant increase was found considering Carnaroli rice and Vialone Nano rice.

However, comparing boiled rice varieties, Basmati rice and Carnaroli rice showed the highest percentage of amylose with respect to total carbohydrates (39.22 ± 0.41; 39.83 ± 0.86; respectively), both statistically different from the other rice varieties. On the contrary, Arborio rice showed the lowest percentage of amylose (27.80 ± 0.55) with respect to total carbohydrates, statistically significant different from all the others.

In case of stewing, it was possible to observe a general increase in terms of percentage of amylose with respect to total carbohydrates with the exception of Ribe rice in which the percentage is maintained and Carnaroli rice in which it significantly decreased. Basmati rice resulted again to have the highest percentage of amylose with respect to total carbohydrates (45.06 ± 0.96) significantly different from all the other rice varieties except for Arborio rice that, in this case, showed one on the highest percentage of amylose respect to total carbohydrates (44.36 ± 0.39). The lowest value was shown by stewed Carnaroli rice (25.75 ± 0.44).

Microwaving cooking process did not alter percentage of amylose with respect to total carbohydrates compared to raw products in Carnaroli, Fragrance and Arborio rice. The only rice variety which showed a significant increase in the percentage of amylose with respect to total carbohydrates was Vialone Nano while Ribe and Basmati rice underwent a decrease.

Comparing the percentage of amylose with respect to total carbohydrates among microwaved rice varieties, Basmati rice again showed one of the highest values with Fragrance rice and Arborio rice (36.77 ± 0.13; 37.26 ± 0.40; 35.87 ± 0.16; respectively). As in stewing, Carnaroli rice showed the lowest percentage of amylose with respect to total carbohydrates (30.80 ± 0.26). Multiple comparisons are reported in Table S4.

Therefore, the percentage of amylose with respect to total carbohydrates was associated to both rice variety and cooking method. Indeed, from the obtained results, it was possible to observe that each rice variety can show either an increase or a decrease in percentage of amylose with respect to total carbohydrates depending on cooking method.

## 4. Discussion

This study underlined the differences in nutritional characteristics of rice varieties and, particularly, the effects of cooking methods on their nutritional quality. Obtained data prove to be of remarkable interest considering the importance of varying foods for health and the frequent use of rice in gluten free diets [36,42,43].

Differences have emerged in terms of protein, total carbohydrates and amylose content among the six analyzed rice varieties (Ribe rice, Basmati rice, Carnaroli rice, Vialone Nano rice, Fragrance rice and Arborio rice) both as raw product and after cooking (boiling, stewing and microwaving).

Protein content of analysed raw rice (ranged from 5.89 to 8.71 g/100 g) was in line with ranges reported by other studies 6–12 g/100 g [42,44] or 7–9 g/100 g [45]. Differences in terms of protein content have emerged considering either rice variety or cooking method in accordance with data reported in literature [42]. In particular, according to Khatoon et al., the protein content of different rice varieties changes depending on the type of rice considered and the cooking method applied [42].

By the analysis of obtained data from the present study, all cooking methods in general induced a decrease in protein content with boiling showing the highest effect, followed by stewing and microwaving.

Moreover, carbohydrate content is modified after cooking [36,46]. In particular, in the study of Adi et al., total carbohydrates show a reduction after different cooking methods application (conventional cooking, rice cooker and steaming) in all the analysed rice varieties (Black rice, Brown rice, semi-organic white rice and organic white rice) [36]. The carbohydrate content of the six evaluated rice varieties was around 76% with no significant differences among raw rice varieties and close to percentages reported in literature (about 80%) [47]. However, remarkable differences were found among rice considering both the variety of rice and the cooking method used. In particular, boiling caused the highest loss in terms of total carbohydrates, compared to the other cooking methods considered, followed by stewing. On the contrary, microwaving preserves the highest content of total carbohydrates.

Among carbohydrates, it is interesting to focus the attention on amylose content.

Different rice varieties contain different amounts of amylose, and this content results in diverse nutritional characteristics, health impacts, cooking properties, and organoleptic features of rice [48]. Amylose content influences water absorption, volume expansion and stickiness of rice grains influencing rice sensory properties. Indeed, the higher the amylose content, the lower the stickiness of rice grains [48].

Furthermore, high amylose starch is resistant to digestion and acts as dietary fiber [22,26,49,50,51]. Dietary fiber is associated with many health benefits as lower glycaemic index, promoting satiety and acting as prebiotic [26,52]. Thus, amylose content contributes to manage the glycaemic index of the food [53] and postprandial glycaemic response [50]. Glycaemic index is used to measure blood glucose response after the ingestion of carbohydrate-containing foods [54] aiding to evaluate the quality of dietary carbohydrates [55]. This knowledge is particularly interesting from a nutritional point of view because carbohydrate composition can guide product selection in case of specific nutritional needs. In diabetes and metabolic disorders, for example, medium and low glycaemic index foods must be preferred than high glycaemic ones [56]. For this reason, selecting a rice variety with a high amylose content could be a strategy to better manage the glycaemic response after a meal [39].

From the present study, amylose content of the six raw analysed rice varieties ranges between 24.36 and 32.64 g/100 g. According to Acquistucci et al. amylose content varies from 15.5 to 25.2 g/100 g of dry weight [57]. Anyway, it is necessary to consider that amylose content changes depending also on the ambient temperatures during the growth of cereal grains. Indeed, lower ambient temperature during rice development induces an increase in amylose content [58,59].

Considering amylose content, rice varieties can be classified depending on amylose amount in 100 g of product as waxy (0–5%), very low (5–12%), low (12–20%), intermediate (20–25%), and high (25–33%) [60]. Moreover, Suwannaporn et al. found that rice varieties available in the market can be classified according to amylose content in 100 g of products as low (less than 20%amylose), medium (21–25%) or high (26–33%) [61]. According to this, Table 7 shows the classification of the six evaluated rice varieties depending on their amylose content. Since the impact of cooking methods on nutritional quality of cereals has been reported in literature [62,63], it remains of great interest to further exploit this aspect to better study cereals nutritional quality.

In this perspective, Table 7 shows a classification based on amylose content both for raw rice and after the application of the three studied cooking methods.

The six raw rice varieties studied can be classified as medium (Carnaroli rice and Vialone Nano rice) and high (Ribe rice, Basmati rice, Fragrance rice and Arborio rice). However, after cooking processes differences can be found.

The marked loss of amylose due to boiling classified all rice varieties as low. The same table underlines that stewing has a negative impact on amylose content.

Microwaving resulted the cooking method with the less impact in terms of amylose content.

Among rice varieties considered, Basmati rice and Fragrance rice are of interest as they maintain a medium-high classification when stewed or microwaved. Similar results were pointed out by Rahim et al., suggesting a preference for Basmati and brown rice, especially for diabetic patients [64], and by Simonelli et al. regarding Fragrance rice [48].

From obtained data, differences were found in terms of total carbohydrates depending on cooking method. For this reason, amylose content was also evaluated referring to total carbohydrates in order to observe possible changes of percentage of amylose content with respect to total carbohydrates.

In raw products amylose percentage with respect to total carbohydrates varies according to the variety of rice considered. In particular, Basmati rice is the one with the highest percentage. Beyond rice variety, also in this case, it is necessary to consider the specific cooking method applied.

From the obtained results, it may be possible to point out the importance of considering cooking methods impact on nutritional quality of product taking into account not only amylose content but also percentage of amylose with respect to total carbohydrates content.

**Figure 1:**
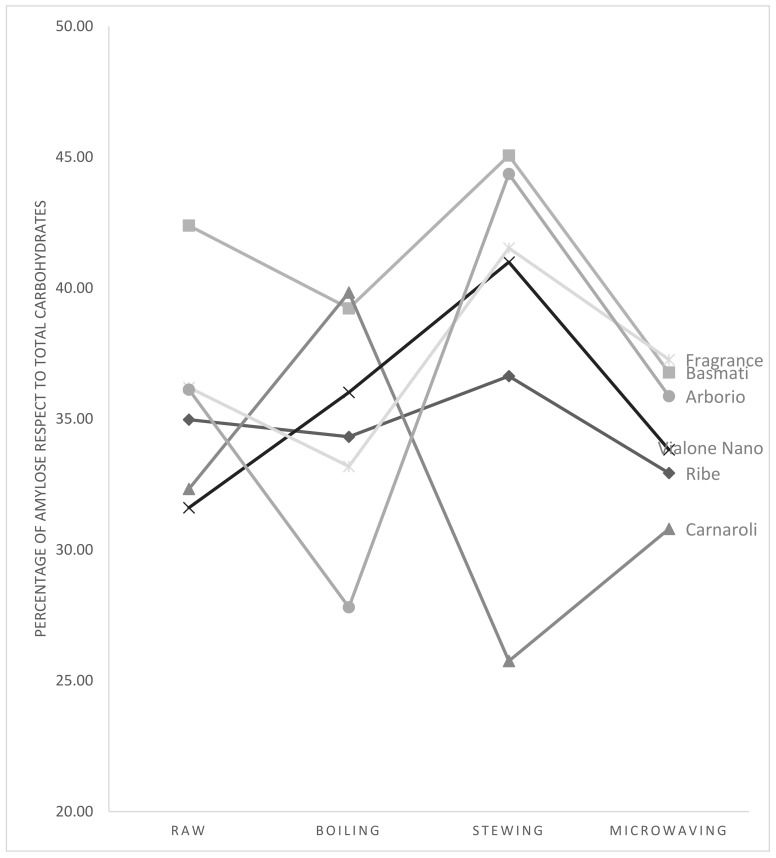
Graphical representation of amylose percentage with respect to total carbohydrate changes depending on cooking methods and rice varieties.

Due to the importance of the percentage of amylose with respect to total carbohydrates from a practical point of view, in Figure 1, a graphical representation of obtained results is proposed to be used as a tool in nutritionist practice.

In particular, considering percentages, generally the highest values were obtained with stewing (above all for Basmati rice and Arborio rice), however exceptions can be underlined as in case of Carnaroli rice in which the highest percentage was associated with boiling.

In this context, the role of amylose content and amylose percentage with respect to total carbohydrates appears to be of great importance to better manage nutritional quality of diet and to focus the attention on glycaemic index of products and, consequently, glycaemic response. For example, in the study, it is shown in that for Basmati rice and Arborio rice, it could be suggested to use stewing in order to improve the percentage of amylose with respect to total carbohydrates. On the contrary, in other contexts, it could be useful to favor cooking methods that induce a reduction of the percentage of amylose with respect to total carbohydrates, in order to increase carbohydrates availability and to contribute to recovery after physical exercise [65,66].

Beside proteins and carbohydrates content of rice, fiber, minerals as manganese, selenium, iron and phosphorus and vitamins as folic acid, thiamine and niacin [67,68] should also be mentioned. However, contribution in nutrient intakes is strictly related to proportion of germ, bran and endosperm [68]. For example, although energy and macronutrients contents are similar between white and brown rice, brown rice contains considerably more fibre and is higher in some minerals, particularly magnesium [68].

As reported in Table 1, the fiber content of the six analyzed rice ranged from 0.7 to 1.9 g of 100 g of raw product =, with Carnaroli rice showing the lowest value, while Arborio rice the highest.

Nutrients, and in particular mineral content, depend not only on rice processing (i.e., polishing, milling, and parboiling), but are also influenced by rice variety (i.e., differences can be found in terms of potassium, phosphorus and magnesium among long grain rice, brown rice, white basmati, and wholegrain basmati rice) and cooking methods [68,69,70,71]. For example, a study by Mwale and colleagues, reported a significant loss of minerals when rice was cooked using a rice-to-water ratio of 1:6 above all of potassium [71].

Thus, encouraging the use of naturally gluten-free products and varying food choices are fundamental aspects related to the improvement of a GF-diet quality.

Rice is an important staple food for more than half of the world’s population [72], representing one of the main sources of carbohydrate with an important role in a healthy diet [68]. However, it should be considered as part of a balanced and varied diet [12,13]. Indeed, cereals, including rice, should be present at each meal together with healthy proteins, plant-based fat, vegetables, and fruits [34].

## 5. Conclusions

Beside the limitation of the restricted number of rice varieties and cooking methods analysed, results obtained from this study represent an important starting point to guide adequate food and cooking method choices in order to better manage the diet and improve its nutritional quality, especially in specific conditions, like celiac disease. To the authors’ knowledge, there is a lack of literature giving suggestions on how to choose adequate food and cooking methods in the case of celiac patients.

These results can be useful in the practice of nutritionists or experts in nutrition to counsel patients who require specific recommendations.

Moreover, this knowledge can aid consumers, in particular celiac patients, to make healthy and responsible dietary selections to establish a balanced diet.

## Figures and Tables

**Table 1:** Nutritional facts of the six selected raw rice varieties from nutritional labels.

Nutrition Facts (for 100 g of Raw Rice)					
Rice Varieties	Energy (kcal)	Total Fat (g)	Total Carbohydrate (g)	Dietary Fiber (g)	Total Protein (g)
Ribe	350	0.6	76.9	0.9	7.4
Basmati	359	1.3	77.3	1.3	8.8
Carnaroli	348	1.6	75.9	0.7	7.2
Vialone Nano	346	1.1	77.2	1.3	6.2
Fragrance	350	1.2	76.5	1.2	7.8
Arborio	351	1.3	77.0	1.9	6.9

**Table 2:** Cooking time (min) and water (mL) used for each rice variety for the three cooking methods (boiling, stewing and microwaving).

Rice Varieties	Boiling		Stewing		Microwaving	
	Cooking Time (Min)	Water (mL)	Cooking Time (Min)	Water (mL)	Cooking Time (Min)	Water (mL)
Ribe	18	500	18	250	8	166
Basmati	15	500	15	150	8	166
Carnaroli	15	500	15	150	7	166
Vialone Nano	14	500	14	150	7	166
Fragrance	14	500	14	175	8	166
Arborio	14	500	14	175	9	166

**Table 3:** Detectable protein content (g/100 g of rice) of both raw and cooked rice and percentage of retained proteins after the three cooking methods processes.

Rice Varieties		Raw	Boiling	Stewing	Microwaving
Ribe	g/100 g	7.36 ± 0.01	5.73 ± 0.01 *	6.59 ± 0.04 *	7.20 ± 0.01 *
	% ac		77.90 ± 0.26	89.60 ± 0.64	97.89 ± 0.21
Basmati	g/100 g	8.71 ± 0.03	5.55 ± 0.02 *	6.88 ± 0.02 *	7.18 ± 0.01 *
	% ac		63.67 ± 0.40	79.01 ± 0.45	82.45 ± 0.42
Carnaroli	g/100 g	7.14 ± 0.03	5.83 ± 0.01 *	6.41 ± 0.01 *	6.23 ± 0.01 *
	% ac		81.66 ± 0.50	89.69 ± 0.43	87.26 ± 0.39
Vialone Nano	g/100 g	5.89 ± 0.01	5.03 ± 0.01 *	5.66 ± 0.01 *	5.65 ± 0.01 *
	% ac		85.30 ± 0.27	96.10 ± 0.30	95.93 ± 0.30
Fragrance	g/100 g	7.70 ± 0.03	6.06 ± 0.01 *	6.43 ± 0.01 *	6.26 ± 0.01 *
	% ac		78.80 ± 0.46	83.52 ± 0.41	81.40 ± 0.41
Arborio	g/100 g	6.71 ± 0.02	5.88 ± 0.01 *	6.12 ± 0.01 *	6.07 ± 0.01 *
	% ac		87.70 ± 0.38	91.23 ± 0.45	90.48 ± 0.33

Value reported as mean ± sd; % ac = percentage of remained protein content after cooking treatment; * statistically different from raw (*p* < 0.05). Multiple comparisons of different rice and different cooking methods are shown in Table S1.

**Table 4:** Total carbohydrates content (g/100 g) of both raw and cooked rice and percentage of retained total carbohydrates after the three cooking methods processes.

Rice Varieties		Raw	Boiling	Stewing	Microwaving
Ribe	g/100 g	76.23 ± 0.03	40.42 ± 2.99 *	50.03 ± 0.11 *	68.80 ± 0.06 *
	% ac		53.02 ± 3.94	65.63 ± 0.11	90.26 ± 0.05
Basmati	g/100 g	77.01 ± 0.01	49.32 ± 0.58 *	51.62 ± 0.34 *	70.33 ± 0.23 *
	% ac		64.05 ± 0.75	67.04 ± 0.44	91.32 ± 0.28
Carnaroli	g/100 g	75.51 ± 0.01	38.65 ± 0.58 *	67.54 ± 0.24 *	70.61 ± 0.28 *
	% ac		51.18 ± 0.76	89.45 ± 0.30	93.51 ± 0.38
Vialone Nano	g/100 g	77.06 ± 0.01	36.72 ± 0.23 *	43.04 ± 0.43 *	64.52 ± 0.37 *
	% ac		47.64 ± 0.30	55.85 ± 0.56	83.72 ± 0.48
Fragrance	g/100 g	76.01 ± 0.01	37.26 ± 0.85 *	56.48 ± 0.42 *	68.65 ± 0.07 *
	% ac		49.03 ± 1.11	74.31 ± 0.56	90.31 ± 0.11
Arborio	g/100 g	76.86 ± 0.04	41.95 ± 0.80 *	52.70 ± 0.26 *	70.21 ± 0.23 *
	% ac		54.58 ± 1.01	68.56 ± 0.37	91.35 ± 0.27

Value reported as mean ± sd; % ac = percentage of remained total carbohydrates content after cooking treatment; * statistically different from raw (*p* < 0.05). Multiple comparisons of different rice and different cooking methods are shown in Table S2.

**Table 5:** Amylose content (g/100 g) of both raw and cooked rice and percentage of retained amylose after the three cooking methods processes.

Rice Varieties		Raw	Boiling	Stewing	Microwaving
Ribe	g/100 g	26.66 ± 0.01	13.83 ± 0.14 *	18.33 ± 0.19 *	22.66 ± 0.15 *
	% ac		51.87 ± 0.53	68.76 ± 0.69	85.00 ± 0.53
Basmati	g/100 g	32.64 ± 0.02	19.34 ± 0.29 *	23.26 ± 0.42 *	25.86 ± 0.04 *
	% ac		59.27 ± 0.86	71.27 ± 1.25	79.23 ± 0.08
Carnaroli	g/100 g	24.41 ± 0.02	15.40 ± 0.53 *	17.39 ± 0.26 *	21.75 ± 0.15 *
	% ac		63.07 ± 2.17	71.26 ± 1.02	89.09 ± 0.65
Vialone Nano	g/100 g	24.36 ± 0.01	13.22 ± 0.09 *	17.64 ± 0.35 *	21.82 ± 0.13 *
	% ac		54.28 ± 0.36	72.42 ± 1.45	89.58 ± 0.50
Fragrance	g/100 g	27.51 ± 0.02	12.36 ± 0.01 *	23.46 ± 0.37 *	25.58 ± 0.27 *
	% ac		44.93 ± 0.06	85.25 ± 1.28	92.97 ± 0.92
Arborio	g/100 g	27.76 ± 0.02	11.66 ± 0.16 *	23.37 ± 0.27 *	25.18 ± 0.15 *
	% ac		42.01 ± 0.56	84.20 ± 0.94	90.71 ± 0.45

Value reported as mean ± sd; % ac = percentage of remained amylose after cooking treatment; * statistically different from raw (*p* < 0.05). Multiple comparisons of different rice and different cooking methods are shown in Table S3.

**Table 6:** Amylose percentage respect to total carbohydrates in raw rice and after the three cooking methods application.

Rice Varieties	Raw	Boiling	Stewing	Microwaving
Ribe	34.97 ± 0.01	34.32 ± 2.11	36.64 ± 0.32	32.93 ± 0.19 *
Basmati	42.38 ± 0.02	39.22 ± 0.41 *	45.06 ± 0.96 *	36.77 ± 0.13 *
Carnaroli	32.32 ± 0.02	39.83 ± 0.86 *	25.75 ± 0.44 *	30.80 ± 0.26
Vialone Nano	31.61 ± 0.01	36.01 ± 0.37 *	40.99 ± 1.15 *	33.82 ± 0.15 *
Fragrance	36.20 ± 0.04	33.19 ± 0.74 *	41.53 ± 0.55 *	37.26 ± 0.40
Arborio	36.12 ± 0.01	27.80 ± 0.55 *	44.36 ± 0.39 *	35.87 ± 0.16

Value reported as mean ± sd; * statistically different from raw (*p* < 0.05). Multiple comparisons of different rice and different cooking methods are shown in Table S4.

**Table 7:** Rice classification depending on amylose content of the six analyzed rice varieties as raw and after the application of the three considered cooking methods (according to Suwannaporn et al. classification for raw rice).

Rice Type	Raw	Boiling	Stewing	Microwaving
Ribe	High	Low	Low	Medium
Basmati	High	Low	Medium	High
Carnaroli	Medium	Low	Low	Medium
Vialone Nano	Medium	Low	Low	Medium
Fragrance	High	Low	Medium	High
Arborio	High	Low	Medium	Medium

## Data Availability

The data presented in this study are available on request from the corresponding author.

## Supplementary Materials

The following are available online at https://www.mdpi.com/article/10.3390/foods10061375/s1, Table S1 Tukey’s multiple comparisons test of detectable proteins content; Table S2. Tukey’s multiple comparisons test of detectable carbohydrates content; Table S3. Tukey’s multiple comparisons test of detectable amylose content; Table S4. Tukey’s multiple comparisons test of detectable amylose/total CHO content after cooking processes.
