# Supplementary material for: Nutritional Properties of Rice Varieties Commonly Consumed in Italy and Applicability in Gluten Free Diet
Source: Foods. 2021 Jun 14;10(6):1375. doi: 10.3390/foods10061375 (PMC8232128; doi:10.3390/foods10061375)
Supplement: Supplementary file 1 [file foods-10-01375-s001.zip › Supplementary Materials 1 Flora.pdf]

Table S1 Tukey's multiple comparisons test of detectable proteins content

Tukey's multiple comparisons test	Summary	Adjusted P Value
Ribe:Raw vs. Ribe:Boiling	****	<0,0001
Ribe:Raw vs. Ribe:Stewing	****	<0,0001
Ribe:Raw vs. Ribe:Microwaving	****	<0,0001
Ribe:Raw vs. Basmati:Raw	****	<0,0001
Ribe:Raw vs. Basmati:Boiling	****	<0,0001
Ribe:Raw vs. Basmati:Stewing	****	<0,0001
Ribe:Raw vs. Basmati:Microwaving	****	<0,0001
Ribe:Raw vs. Carnaroli:Raw	****	<0,0001
Ribe:Raw vs. Carnaroli:Boiling	****	<0,0001
Ribe:Raw vs. Carnaroli:Stewing	****	<0,0001
Ribe:Raw vs. Carnaroli:Microwaving	****	<0,0001
Ribe:Raw vs. Vialone nano:Raw	****	<0,0001
Ribe:Raw vs. Vialone nano:Boiling	****	<0,0001
Ribe:Raw vs. Vialone nano:Stewing	****	<0,0001
Ribe:Raw vs. Vialone nano:Microwaving	****	<0,0001
Ribe:Raw vs. Fragrance:Raw	****	<0,0001
Ribe:Raw vs. Fragrance:Boiling	****	<0,0001
Ribe:Raw vs. Fragrance:Stewing	****	<0,0001
Ribe:Raw vs. Fragrance:Microwaving	****	<0,0001
Ribe:Raw vs. Arborio:Raw	****	<0,0001
Ribe:Raw vs. Arborio:Boiling	****	<0,0001
Ribe:Raw vs. Arborio:Stewing	****	<0,0001
Ribe:Raw vs. Arborio:Microwaving	****	<0,0001
Ribe:Boiling vs. Ribe:Stewing	****	<0,0001
Ribe:Boiling vs. Ribe:Microwaving	****	<0,0001
Ribe:Boiling vs. Basmati:Raw	****	<0,0001
Ribe:Boiling vs. Basmati:Boiling	****	<0,0001
Ribe:Boiling vs. Basmati:Stewing	****	<0,0001
Ribe:Boiling vs. Basmati:Microwaving	****	<0,0001
Ribe:Boiling vs. Carnaroli:Raw	****	<0,0001
Ribe:Boiling vs. Carnaroli:Boiling	****	<0,0001
Ribe:Boiling vs. Carnaroli:Stewing	****	<0,0001
Ribe:Boiling vs. Carnaroli:Microwaving	****	<0,0001
Ribe:Boiling vs. Vialone nano:Raw	****	<0,0001
Ribe:Boiling vs. Vialone nano:Boiling	****	<0,0001
Ribe:Boiling vs. Vialone nano:Stewing	**	0,0029
Ribe:Boiling vs. Vialone nano:Microwaving	***	0,0003
Ribe:Boiling vs. Fragrance:Raw	****	<0,0001
Ribe:Boiling vs. Fragrance:Boiling	****	<0,0001
Ribe:Boiling vs. Fragrance:Stewing	****	<0,0001
Ribe:Boiling vs. Fragrance:Microwaving	****	<0,0001
Ribe:Boiling vs. Arborio:Raw	****	<0,0001
Ribe:Boiling vs. Arborio:Boiling	****	<0,0001
Ribe:Boiling vs. Arborio:Stewing	****	<0,0001
Ribe:Boiling vs. Arborio:Microwaving	****	<0,0001
Ribe:Stewing vs. Ribe:Microwaving	****	<0,0001
Ribe:Stewing vs. Basmati:Raw	****	<0,0001
Ribe:Stewing vs. Basmati:Boiling	****	<0,0001
Ribe:Stewing vs. Basmati:Stewing	****	<0,0001
Ribe:Stewing vs. Basmati:Microwaving	****	<0,0001
Ribe:Stewing vs. Carnaroli:Raw	****	<0,0001
Ribe:Stewing vs. Carnaroli:Boiling	****	<0,0001
Ribe:Stewing vs. Carnaroli:Stewing	****	<0,0001
Ribe:Stewing vs. Carnaroli:Microwaving	****	<0,0001
Ribe:Stewing vs. Vialone nano:Raw	****	<0,0001
Ribe:Stewing vs. Vialone nano:Boiling	****	<0,0001
Ribe:Stewing vs. Vialone nano:Stewing	****	<0,0001
Ribe:Stewing vs. Vialone nano:Microwaving	****	<0,0001
Ribe:Stewing vs. Fragrance:Raw	****	<0,0001
Ribe:Stewing vs. Fragrance:Boiling	****	<0,0001

Ribe:Stewing vs. Fragrance:Stewing	****	<0,0001
Ribe:Stewing vs. Fragrance:Microwaving	****	<0,0001
Ribe:Stewing vs. Arborio:Raw	****	<0,0001
Ribe:Stewing vs. Arborio:Boiling	****	<0,0001
Ribe:Stewing vs. Arborio:Stewing	****	<0,0001
Ribe:Stewing vs. Arborio:Microwaving	****	<0,0001
Ribe:Microwaving vs. Basmati:Raw	****	<0,0001
Ribe:Microwaving vs. Basmati:Boiling	****	<0,0001
Ribe:Microwaving vs. Basmati:Stewing	****	<0,0001
Ribe:Microwaving vs. Basmati:Microwaving	ns	0,9999
Ribe:Microwaving vs. Carnaroli:Raw	*	0,0250
Ribe:Microwaving vs. Carnaroli:Boiling	****	<0,0001
Ribe:Microwaving vs. Carnaroli:Stewing	****	<0,0001
Ribe:Microwaving vs. Carnaroli:Microwaving	****	<0,0001
Ribe:Microwaving vs. Vialone nano:Raw	****	<0,0001
Ribe:Microwaving vs. Vialone nano:Boiling	****	<0,0001
Ribe:Microwaving vs. Vialone nano:Stewing	****	<0,0001
Ribe:Microwaving vs. Vialone nano:Microwaving	****	<0,0001
Ribe:Microwaving vs. Fragrance:Raw	****	<0,0001
Ribe:Microwaving vs. Fragrance:Boiling	****	<0,0001
Ribe:Microwaving vs. Fragrance:Stewing	****	<0,0001
Ribe:Microwaving vs. Fragrance:Microwaving	****	<0,0001
Ribe:Microwaving vs. Arborio:Raw	****	<0,0001
Ribe:Microwaving vs. Arborio:Boiling	****	<0,0001
Ribe:Microwaving vs. Arborio:Stewing	****	<0,0001
Ribe:Microwaving vs. Arborio:Microwaving	****	<0,0001
Basmati:Raw vs. Basmati:Boiling	****	<0,0001
Basmati:Raw vs. Basmati:Stewing	****	<0,0001
Basmati:Raw vs. Basmati:Microwaving	****	<0,0001
Basmati:Raw vs. Carnaroli:Raw	****	<0,0001
Basmati:Raw vs. Carnaroli:Boiling	****	<0,0001
Basmati:Raw vs. Carnaroli:Stewing	****	<0,0001
Basmati:Raw vs. Carnaroli:Microwaving	****	<0,0001
Basmati:Raw vs. Vialone nano:Raw	****	<0,0001
Basmati:Raw vs. Vialone nano:Boiling	****	<0,0001
Basmati:Raw vs. Vialone nano:Stewing	****	<0,0001
Basmati:Raw vs. Vialone nano:Microwaving	****	<0,0001
Basmati:Raw vs. Fragrance:Raw	****	<0,0001
Basmati:Raw vs. Fragrance:Boiling	****	<0,0001
Basmati:Raw vs. Fragrance:Stewing	****	<0,0001
Basmati:Raw vs. Fragrance:Microwaving	****	<0,0001
Basmati:Raw vs. Arborio:Raw	****	<0,0001
Basmati:Raw vs. Arborio:Boiling	****	<0,0001
Basmati:Raw vs. Arborio:Stewing	****	<0,0001
Basmati:Raw vs. Arborio:Microwaving	****	<0,0001
Basmati:Boiling vs. Basmati:Stewing	****	<0,0001
Basmati:Boiling vs. Basmati:Microwaving	****	<0,0001
Basmati:Boiling vs. Carnaroli:Raw	****	<0,0001
Basmati:Boiling vs. Carnaroli:Boiling	****	<0,0001
Basmati:Boiling vs. Carnaroli:Stewing	****	<0,0001
Basmati:Boiling vs. Carnaroli:Microwaving	****	<0,0001
Basmati:Boiling vs. Vialone nano:Raw	****	<0,0001
Basmati:Boiling vs. Vialone nano:Boiling	****	<0,0001
Basmati:Boiling vs. Vialone nano:Stewing	****	<0,0001
Basmati:Boiling vs. Vialone nano:Microwaving	****	<0,0001
Basmati:Boiling vs. Fragrance:Raw	****	<0,0001
Basmati:Boiling vs. Fragrance:Boiling	****	<0,0001
Basmati:Boiling vs. Fragrance:Stewing	****	<0,0001
Basmati:Boiling vs. Fragrance:Microwaving	****	<0,0001
Basmati:Boiling vs. Arborio:Raw	****	<0,0001
Basmati:Boiling vs. Arborio:Boiling	****	<0,0001
Basmati:Boiling vs. Arborio:Stewing	****	<0,0001
Basmati:Boiling vs. Arborio:Microwaving	****	<0,0001
Basmati:Stewing vs. Basmati:Microwaving	****	<0,0001

Basmati:Stewing vs. Carnaroli:Raw	****	<0,0001
Basmati:Stewing vs. Carnaroli:Boiling	****	<0,0001
Basmati:Stewing vs. Carnaroli:Stewing	****	<0,0001
Basmati:Stewing vs. Carnaroli:Microwaving	****	<0,0001
Basmati:Stewing vs. Vialone nano:Raw	****	<0,0001
Basmati:Stewing vs. Vialone nano:Boiling	****	<0,0001
Basmati:Stewing vs. Vialone nano:Stewing	****	<0,0001
Basmati:Stewing vs. Vialone nano:Microwaving	****	<0,0001
Basmati:Stewing vs. Fragrance:Raw	****	<0,0001
Basmati:Stewing vs. Fragrance:Boiling	****	<0,0001
Basmati:Stewing vs. Fragrance:Stewing	****	<0,0001
Basmati:Stewing vs. Fragrance:Microwaving	****	<0,0001
Basmati:Stewing vs. Arborio:Raw	****	<0,0001
Basmati:Stewing vs. Arborio:Boiling	****	<0,0001
Basmati:Stewing vs. Arborio:Stewing	****	<0,0001
Basmati:Stewing vs. Arborio:Microwaving	****	<0,0001
Basmati:Microwaving vs. Carnaroli:Raw	ns	0,3932
Basmati:Microwaving vs. Carnaroli:Boiling	****	<0,0001
Basmati:Microwaving vs. Carnaroli:Stewing	****	<0,0001
Basmati:Microwaving vs. Carnaroli:Microwaving	****	<0,0001
Basmati:Microwaving vs. Vialone nano:Raw	****	<0,0001
Basmati:Microwaving vs. Vialone nano:Boiling	****	<0,0001
Basmati:Microwaving vs. Vialone nano:Stewing	****	<0,0001
Basmati:Microwaving vs. Vialone nano:Microwaving	****	<0,0001
Basmati:Microwaving vs. Fragrance:Raw	****	<0,0001
Basmati:Microwaving vs. Fragrance:Boiling	****	<0,0001
Basmati:Microwaving vs. Fragrance:Stewing	****	<0,0001
Basmati:Microwaving vs. Fragrance:Microwaving	****	<0,0001
Basmati:Microwaving vs. Arborio:Raw	****	<0,0001
Basmati:Microwaving vs. Arborio:Boiling	****	<0,0001
Basmati:Microwaving vs. Arborio:Stewing	****	<0,0001
Basmati:Microwaving vs. Arborio:Microwaving	****	<0,0001
Carnaroli:Raw vs. Carnaroli:Boiling	****	<0,0001
Carnaroli:Raw vs. Carnaroli:Stewing	****	<0,0001
Carnaroli:Raw vs. Carnaroli:Microwaving	****	<0,0001
Carnaroli:Raw vs. Vialone nano:Raw	****	<0,0001
Carnaroli:Raw vs. Vialone nano:Boiling	****	<0,0001
Carnaroli:Raw vs. Vialone nano:Stewing	****	<0,0001
Carnaroli:Raw vs. Vialone nano:Microwaving	****	<0,0001
Carnaroli:Raw vs. Fragrance:Raw	****	<0,0001
Carnaroli:Raw vs. Fragrance:Boiling	****	<0,0001
Carnaroli:Raw vs. Fragrance:Stewing	****	<0,0001
Carnaroli:Raw vs. Fragrance:Microwaving	****	<0,0001
Carnaroli:Raw vs. Arborio:Raw	****	<0,0001
Carnaroli:Raw vs. Arborio:Boiling	****	<0,0001
Carnaroli:Raw vs. Arborio:Stewing	****	<0,0001
Carnaroli:Raw vs. Arborio:Microwaving	****	<0,0001
Carnaroli:Boiling vs. Carnaroli:Stewing	****	<0,0001
Carnaroli:Boiling vs. Carnaroli:Microwaving	****	<0,0001
Carnaroli:Boiling vs. Vialone nano:Raw	*	0,0134
Carnaroli:Boiling vs. Vialone nano:Boiling	****	<0,0001
Carnaroli:Boiling vs. Vialone nano:Stewing	****	<0,0001
Carnaroli:Boiling vs. Vialone nano:Microwaving	****	<0,0001
Carnaroli:Boiling vs. Fragrance:Raw	****	<0,0001
Carnaroli:Boiling vs. Fragrance:Boiling	****	<0,0001
Carnaroli:Boiling vs. Fragrance:Stewing	****	<0,0001
Carnaroli:Boiling vs. Fragrance:Microwaving	****	<0,0001
Carnaroli:Boiling vs. Arborio:Raw	****	<0,0001
Carnaroli:Boiling vs. Arborio:Boiling	ns	0,1589
Carnaroli:Boiling vs. Arborio:Stewing	****	<0,0001
Carnaroli:Boiling vs. Arborio:Microwaving	****	<0,0001
Carnaroli:Stewing vs. Carnaroli:Microwaving	****	<0,0001
Carnaroli:Stewing vs. Vialone nano:Raw	****	<0,0001
Carnaroli:Stewing vs. Vialone nano:Boiling	****	<0,0001

Carnaroli:Stewing vs. Vialone nano:Stewing	****	<0,0001
Carnaroli:Stewing vs. Vialone nano:Microwaving	****	<0,0001
Carnaroli:Stewing vs. Fragrance:Raw	****	<0,0001
Carnaroli:Stewing vs. Fragrance:Boiling	****	<0,0001
Carnaroli:Stewing vs. Fragrance:Stewing	ns	0,9982
Carnaroli:Stewing vs. Fragrance:Microwaving	****	<0,0001
Carnaroli:Stewing vs. Arborio:Raw	****	<0,0001
Carnaroli:Stewing vs. Arborio:Boiling	****	<0,0001
Carnaroli:Stewing vs. Arborio:Stewing	****	<0,0001
Carnaroli:Stewing vs. Arborio:Microwaving	****	<0,0001
Carnaroli:Microwaving vs. Vialone nano:Raw	****	<0,0001
Carnaroli:Microwaving vs. Vialone nano:Boiling	****	<0,0001
Carnaroli:Microwaving vs. Vialone nano:Stewing	****	<0,0001
Carnaroli:Microwaving vs. Vialone nano:Microwaving	****	<0,0001
Carnaroli:Microwaving vs. Fragrance:Raw	****	<0,0001
Carnaroli:Microwaving vs. Fragrance:Boiling	****	<0,0001
Carnaroli:Microwaving vs. Fragrance:Stewing	****	<0,0001
Carnaroli:Microwaving vs. Fragrance:Microwaving	ns	0,8647
Carnaroli:Microwaving vs. Arborio:Raw	****	<0,0001
Carnaroli:Microwaving vs. Arborio:Boiling	****	<0,0001
Carnaroli:Microwaving vs. Arborio:Stewing	****	<0,0001
Carnaroli:Microwaving vs. Arborio:Microwaving	****	<0,0001
Vialone nano:Raw vs. Vialone nano:Boiling	****	<0,0001
Vialone nano:Raw vs. Vialone nano:Stewing	****	<0,0001
Vialone nano:Raw vs. Vialone nano:Microwaving	****	<0,0001
Vialone nano:Raw vs. Fragrance:Raw	****	<0,0001
Vialone nano:Raw vs. Fragrance:Boiling	****	<0,0001
Vialone nano:Raw vs. Fragrance:Stewing	****	<0,0001
Vialone nano:Raw vs. Fragrance:Microwaving	****	<0,0001
Vialone nano:Raw vs. Arborio:Raw	****	<0,0001
Vialone nano:Raw vs. Arborio:Boiling	ns	>0,9999
Vialone nano:Raw vs. Arborio:Stewing	****	<0,0001
Vialone nano:Raw vs. Arborio:Microwaving	****	<0,0001
Vialone nano:Boiling vs. Vialone nano:Stewing	****	<0,0001
Vialone nano:Boiling vs. Vialone nano:Microwaving	****	<0,0001
Vialone nano:Boiling vs. Fragrance:Raw	****	<0,0001
Vialone nano:Boiling vs. Fragrance:Boiling	****	<0,0001
Vialone nano:Boiling vs. Fragrance:Stewing	****	<0,0001
Vialone nano:Boiling vs. Fragrance:Microwaving	****	<0,0001
Vialone nano:Boiling vs. Arborio:Raw	****	<0,0001
Vialone nano:Boiling vs. Arborio:Boiling	****	<0,0001
Vialone nano:Boiling vs. Arborio:Stewing	****	<0,0001
Vialone nano:Boiling vs. Arborio:Microwaving	****	<0,0001
Vialone nano:Stewing vs. Vialone nano:Microwaving	ns	>0,9999
Vialone nano:Stewing vs. Fragrance:Raw	****	<0,0001
Vialone nano:Stewing vs. Fragrance:Boiling	****	<0,0001
Vialone nano:Stewing vs. Fragrance:Stewing	****	<0,0001
Vialone nano:Stewing vs. Fragrance:Microwaving	****	<0,0001
Vialone nano:Stewing vs. Arborio:Raw	****	<0,0001
Vialone nano:Stewing vs. Arborio:Boiling	****	<0,0001
Vialone nano:Stewing vs. Arborio:Stewing	****	<0,0001
Vialone nano:Stewing vs. Arborio:Microwaving	****	<0,0001
Vialone nano:Microwaving vs. Fragrance:Raw	****	<0,0001
Vialone nano:Microwaving vs. Fragrance:Boiling	****	<0,0001
Vialone nano:Microwaving vs. Fragrance:Stewing	****	<0,0001
Vialone nano:Microwaving vs. Fragrance:Microwaving	****	<0,0001
Vialone nano:Microwaving vs. Arborio:Raw	****	<0,0001
Vialone nano:Microwaving vs. Arborio:Boiling	****	<0,0001
Vialone nano:Microwaving vs. Arborio:Stewing	****	<0,0001
Vialone nano:Microwaving vs. Arborio:Microwaving	****	<0,0001
Fragrance:Raw vs. Fragrance:Boiling	****	<0,0001
Fragrance:Raw vs. Fragrance:Stewing	****	<0,0001
Fragrance:Raw vs. Fragrance:Microwaving	****	<0,0001
Fragrance:Raw vs. Arborio:Raw	****	<0,0001

Fragrance:Raw vs. Arborio:Boiling	****	<0,0001
Fragrance:Raw vs. Arborio:Stewing	****	<0,0001
Fragrance:Raw vs. Arborio:Microwaving	****	<0,0001
Fragrance:Boiling vs. Fragrance:Stewing	****	<0,0001
Fragrance:Boiling vs. Fragrance:Microwaving	****	<0,0001
Fragrance:Boiling vs. Arborio:Raw	****	<0,0001
Fragrance:Boiling vs. Arborio:Boiling	****	<0,0001
Fragrance:Boiling vs. Arborio:Stewing	*	0,0494
Fragrance:Boiling vs. Arborio:Microwaving	ns	>0,9999
Fragrance:Stewing vs. Fragrance:Microwaving	****	<0,0001
Fragrance:Stewing vs. Arborio:Raw	****	<0,0001
Fragrance:Stewing vs. Arborio:Boiling	****	<0,0001
Fragrance:Stewing vs. Arborio:Stewing	****	<0,0001
Fragrance:Stewing vs. Arborio:Microwaving	****	<0,0001
Fragrance:Microwaving vs. Arborio:Raw	****	<0,0001
Fragrance:Microwaving vs. Arborio:Boiling	****	<0,0001
Fragrance:Microwaving vs. Arborio:Stewing	****	<0,0001
Fragrance:Microwaving vs. Arborio:Microwaving	****	<0,0001
Arborio:Raw vs. Arborio:Boiling	****	<0,0001
Arborio:Raw vs. Arborio:Stewing	****	<0,0001
Arborio:Raw vs. Arborio:Microwaving	****	<0,0001
Arborio:Boiling vs. Arborio:Stewing	****	<0,0001
Arborio:Boiling vs. Arborio:Microwaving	****	<0,0001
Arborio:Stewing vs. Arborio:Microwaving	ns	0,0909
