# Supplementary material for: Nutritional Properties of Rice Varieties Commonly Consumed in Italy and Applicability in Gluten Free Diet
Source: Foods. 2021 Jun 14;10(6):1375. doi: 10.3390/foods10061375 (PMC8232128; doi:10.3390/foods10061375)
Supplement: Supplementary file 1 [file foods-10-01375-s001.zip › Supplementary Materials 2 Flora.pdf]

Table S2. Tukey's multiple comparisons test of detectable carbohydrates content.

Tukey's multiple comparisons test	Summary	Adjusted P Value
Ribe:Raw vs. Ribe:Boiling	****	<0,0001
Ribe:Raw vs. Ribe:Stewing	****	<0,0001
Ribe:Raw vs. Ribe:Microwaving	****	<0,0001
Ribe:Raw vs. Basmati:Raw	ns	0,9993
Ribe:Raw vs. Basmati:Boiling	****	<0,0001
Ribe:Raw vs. Basmati:Stewing	****	<0,0001
Ribe:Raw vs. Basmati:Microwaving	****	<0,0001
Ribe:Raw vs. Carnaroli:Raw	ns	0,9998
Ribe:Raw vs. Carnaroli:Boiling	****	<0,0001
Ribe:Raw vs. Carnaroli:Stewing	****	<0,0001
Ribe:Raw vs. Carnaroli:Microwaving	****	<0,0001
Ribe:Raw vs. Vialone nano:Raw	ns	0,9982
Ribe:Raw vs. Vialone nano:Boiling	****	<0,0001
Ribe:Raw vs. Vialone nano:Stewing	****	<0,0001
Ribe:Raw vs. Vialone nano:Microwaving	****	<0,0001
Ribe:Raw vs. Fragrance:Raw	ns	>0,9999
Ribe:Raw vs. Fragrance:Boiling	****	<0,0001
Ribe:Raw vs. Fragrance:Stewing	****	<0,0001
Ribe:Raw vs. Fragrance:Microwaving	****	<0,0001
Ribe:Raw vs. Arborio:Raw	ns	>0,9999
Ribe:Raw vs. Arborio:Boiling	****	<0,0001
Ribe:Raw vs. Arborio:Stewing	****	<0,0001
Ribe:Raw vs. Arborio:Microwaving	****	<0,0001
Ribe:Boiling vs. Ribe:Stewing	****	<0,0001
Ribe:Boiling vs. Ribe:Microwaving	****	<0,0001
Ribe:Boiling vs. Basmati:Raw	****	<0,0001
Ribe:Boiling vs. Basmati:Boiling	****	<0,0001
Ribe:Boiling vs. Basmati:Stewing	****	<0,0001
Ribe:Boiling vs. Basmati:Microwaving	****	<0,0001
Ribe:Boiling vs. Carnaroli:Raw	****	<0,0001
Ribe:Boiling vs. Carnaroli:Boiling	ns	0,2959
Ribe:Boiling vs. Carnaroli:Stewing	****	<0,0001
Ribe:Boiling vs. Carnaroli:Microwaving	****	<0,0001
Ribe:Boiling vs. Vialone nano:Raw	****	<0,0001
Ribe:Boiling vs. Vialone nano:Boiling	****	<0,0001
Ribe:Boiling vs. Vialone nano:Stewing	**	0,0068
Ribe:Boiling vs. Vialone nano:Microwaving	****	<0,0001
Ribe:Boiling vs. Fragrance:Raw	****	<0,0001
Ribe:Boiling vs. Fragrance:Boiling	***	0,0004
Ribe:Boiling vs. Fragrance:Stewing	****	<0,0001
Ribe:Boiling vs. Fragrance:Microwaving	****	<0,0001
Ribe:Boiling vs. Arborio:Raw	****	<0,0001
Ribe:Boiling vs. Arborio:Boiling	ns	0,5632
Ribe:Boiling vs. Arborio:Stewing	****	<0,0001
Ribe:Boiling vs. Arborio:Microwaving	****	<0,0001
Ribe:Stewing vs. Ribe:Microwaving	****	<0,0001
Ribe:Stewing vs. Basmati:Raw	****	<0,0001
Ribe:Stewing vs. Basmati:Boiling	ns	0,9998
Ribe:Stewing vs. Basmati:Stewing	ns	0,4878
Ribe:Stewing vs. Basmati:Microwaving	****	<0,0001
Ribe:Stewing vs. Carnaroli:Raw	****	<0,0001
Ribe:Stewing vs. Carnaroli:Boiling	****	<0,0001
Ribe:Stewing vs. Carnaroli:Stewing	****	<0,0001
Ribe:Stewing vs. Carnaroli:Microwaving	****	<0,0001
Ribe:Stewing vs. Vialone nano:Raw	****	<0,0001
Ribe:Stewing vs. Vialone nano:Boiling	****	<0,0001
Ribe:Stewing vs. Vialone nano:Stewing	****	<0,0001
Ribe:Stewing vs. Vialone nano:Microwaving	****	<0,0001
Ribe:Stewing vs. Fragrance:Raw	****	<0,0001
Ribe:Stewing vs. Fragrance:Boiling	****	<0,0001

Ribe:Stewing vs. Fragrance:Stewing	****	<0,0001
Ribe:Stewing vs. Fragrance:Microwaving	****	<0,0001
Ribe:Stewing vs. Arborio:Raw	****	<0,0001
Ribe:Stewing vs. Arborio:Boiling	****	<0,0001
Ribe:Stewing vs. Arborio:Stewing	**	0,0056
Ribe:Stewing vs. Arborio:Microwaving	****	<0,0001
Ribe:Microwaving vs. Basmati:Raw	****	<0,0001
Ribe:Microwaving vs. Basmati:Boiling	****	<0,0001
Ribe:Microwaving vs. Basmati:Stewing	****	<0,0001
Ribe:Microwaving vs. Basmati:Microwaving	ns	0,5745
Ribe:Microwaving vs. Carnaroli:Raw	****	<0,0001
Ribe:Microwaving vs. Carnaroli:Boiling	****	<0,0001
Ribe:Microwaving vs. Carnaroli:Stewing	ns	0,8544
Ribe:Microwaving vs. Carnaroli:Microwaving	ns	0,2646
Ribe:Microwaving vs. Vialone nano:Raw	****	<0,0001
Ribe:Microwaving vs. Vialone nano:Boiling	****	<0,0001
Ribe:Microwaving vs. Vialone nano:Stewing	****	<0,0001
Ribe:Microwaving vs. Vialone nano:Microwaving	****	<0,0001
Ribe:Microwaving vs. Fragrance:Raw	****	<0,0001
Ribe:Microwaving vs. Fragrance:Boiling	****	<0,0001
Ribe:Microwaving vs. Fragrance:Stewing	****	<0,0001
Ribe:Microwaving vs. Fragrance:Microwaving	ns	>0,9999
Ribe:Microwaving vs. Arborio:Raw	****	<0,0001
Ribe:Microwaving vs. Arborio:Boiling	****	<0,0001
Ribe:Microwaving vs. Arborio:Stewing	****	<0,0001
Ribe:Microwaving vs. Arborio:Microwaving	ns	0,7167
Basmati:Raw vs. Basmati:Boiling	****	<0,0001
Basmati:Raw vs. Basmati:Stewing	****	<0,0001
Basmati:Raw vs. Basmati:Microwaving	****	<0,0001
Basmati:Raw vs. Carnaroli:Raw	ns	0,6004
Basmati:Raw vs. Carnaroli:Boiling	****	<0,0001
Basmati:Raw vs. Carnaroli:Stewing	****	<0,0001
Basmati:Raw vs. Carnaroli:Microwaving	****	<0,0001
Basmati:Raw vs. Vialone nano:Raw	ns	>0,9999
Basmati:Raw vs. Vialone nano:Boiling	****	<0,0001
Basmati:Raw vs. Vialone nano:Stewing	****	<0,0001
Basmati:Raw vs. Vialone nano:Microwaving	****	<0,0001
Basmati:Raw vs. Fragrance:Raw	ns	0,9831
Basmati:Raw vs. Fragrance:Boiling	****	<0,0001
Basmati:Raw vs. Fragrance:Stewing	****	<0,0001
Basmati:Raw vs. Fragrance:Microwaving	****	<0,0001
Basmati:Raw vs. Arborio:Raw	ns	>0,9999
Basmati:Raw vs. Arborio:Boiling	****	<0,0001
Basmati:Raw vs. Arborio:Stewing	****	<0,0001
Basmati:Raw vs. Arborio:Microwaving	****	<0,0001
Basmati:Boiling vs. Basmati:Stewing	*	0,0343
Basmati:Boiling vs. Basmati:Microwaving	****	<0,0001
Basmati:Boiling vs. Carnaroli:Raw	****	<0,0001
Basmati:Boiling vs. Carnaroli:Boiling	****	<0,0001
Basmati:Boiling vs. Carnaroli:Stewing	****	<0,0001
Basmati:Boiling vs. Carnaroli:Microwaving	****	<0,0001
Basmati:Boiling vs. Vialone nano:Raw	****	<0,0001
Basmati:Boiling vs. Vialone nano:Boiling	****	<0,0001
Basmati:Boiling vs. Vialone nano:Stewing	****	<0,0001
Basmati:Boiling vs. Vialone nano:Microwaving	****	<0,0001
Basmati:Boiling vs. Fragrance:Raw	****	<0,0001
Basmati:Boiling vs. Fragrance:Boiling	****	<0,0001
Basmati:Boiling vs. Fragrance:Stewing	****	<0,0001
Basmati:Boiling vs. Fragrance:Microwaving	****	<0,0001
Basmati:Boiling vs. Arborio:Raw	****	<0,0001
Basmati:Boiling vs. Arborio:Boiling	****	<0,0001
Basmati:Boiling vs. Arborio:Stewing	****	<0,0001
Basmati:Boiling vs. Arborio:Microwaving	****	<0,0001
Basmati:Stewing vs. Basmati:Microwaving	****	<0,0001

Basmati:Stewing vs. Carnaroli:Raw	****	<0,0001
Basmati:Stewing vs. Carnaroli:Boiling	****	<0,0001
Basmati:Stewing vs. Carnaroli:Stewing	****	<0,0001
Basmati:Stewing vs. Carnaroli:Microwaving	****	<0,0001
Basmati:Stewing vs. Vialone nano:Raw	****	<0,0001
Basmati:Stewing vs. Vialone nano:Boiling	****	<0,0001
Basmati:Stewing vs. Vialone nano:Stewing	****	<0,0001
Basmati:Stewing vs. Vialone nano:Microwaving	****	<0,0001
Basmati:Stewing vs. Fragrance:Raw	****	<0,0001
Basmati:Stewing vs. Fragrance:Boiling	****	<0,0001
Basmati:Stewing vs. Fragrance:Stewing	****	<0,0001
Basmati:Stewing vs. Fragrance:Microwaving	****	<0,0001
Basmati:Stewing vs. Arborio:Raw	****	<0,0001
Basmati:Stewing vs. Arborio:Boiling	****	<0,0001
Basmati:Stewing vs. Arborio:Stewing	ns	0,9652
Basmati:Stewing vs. Arborio:Microwaving	****	<0,0001
Basmati:Microwaving vs. Carnaroli:Raw	****	<0,0001
Basmati:Microwaving vs. Carnaroli:Boiling	****	<0,0001
Basmati:Microwaving vs. Carnaroli:Stewing	**	0,0029
Basmati:Microwaving vs. Carnaroli:Microwaving	ns	>0,9999
Basmati:Microwaving vs. Vialone nano:Raw	****	<0,0001
Basmati:Microwaving vs. Vialone nano:Boiling	****	<0,0001
Basmati:Microwaving vs. Vialone nano:Stewing	****	<0,0001
Basmati:Microwaving vs. Vialone nano:Microwaving	****	<0,0001
Basmati:Microwaving vs. Fragrance:Raw	****	<0,0001
Basmati:Microwaving vs. Fragrance:Boiling	****	<0,0001
Basmati:Microwaving vs. Fragrance:Stewing	****	<0,0001
Basmati:Microwaving vs. Fragrance:Microwaving	ns	0,3875
Basmati:Microwaving vs. Arborio:Raw	****	<0,0001
Basmati:Microwaving vs. Arborio:Boiling	****	<0,0001
Basmati:Microwaving vs. Arborio:Stewing	****	<0,0001
Basmati:Microwaving vs. Arborio:Microwaving	ns	>0,9999
Carnaroli:Raw vs. Carnaroli:Boiling	****	<0,0001
Carnaroli:Raw vs. Carnaroli:Stewing	****	<0,0001
Carnaroli:Raw vs. Carnaroli:Microwaving	****	<0,0001
Carnaroli:Raw vs. Vialone nano:Raw	ns	0,5325
Carnaroli:Raw vs. Vialone nano:Boiling	****	<0,0001
Carnaroli:Raw vs. Vialone nano:Stewing	****	<0,0001
Carnaroli:Raw vs. Vialone nano:Microwaving	****	<0,0001
Carnaroli:Raw vs. Fragrance:Raw	ns	>0,9999
Carnaroli:Raw vs. Fragrance:Boiling	****	<0,0001
Carnaroli:Raw vs. Fragrance:Stewing	****	<0,0001
Carnaroli:Raw vs. Fragrance:Microwaving	****	<0,0001
Carnaroli:Raw vs. Arborio:Raw	ns	0,7744
Carnaroli:Raw vs. Arborio:Boiling	****	<0,0001
Carnaroli:Raw vs. Arborio:Stewing	****	<0,0001
Carnaroli:Raw vs. Arborio:Microwaving	****	<0,0001
Carnaroli:Boiling vs. Carnaroli:Stewing	****	<0,0001
Carnaroli:Boiling vs. Carnaroli:Microwaving	****	<0,0001
Carnaroli:Boiling vs. Vialone nano:Raw	****	<0,0001
Carnaroli:Boiling vs. Vialone nano:Boiling	ns	0,1697
Carnaroli:Boiling vs. Vialone nano:Stewing	****	<0,0001
Carnaroli:Boiling vs. Vialone nano:Microwaving	****	<0,0001
Carnaroli:Boiling vs. Fragrance:Raw	****	<0,0001
Carnaroli:Boiling vs. Fragrance:Boiling	ns	0,7374
Carnaroli:Boiling vs. Fragrance:Stewing	****	<0,0001
Carnaroli:Boiling vs. Fragrance:Microwaving	****	<0,0001
Carnaroli:Boiling vs. Arborio:Raw	****	<0,0001
Carnaroli:Boiling vs. Arborio:Boiling	***	0,0002
Carnaroli:Boiling vs. Arborio:Stewing	****	<0,0001
Carnaroli:Boiling vs. Arborio:Microwaving	****	<0,0001
Carnaroli:Stewing vs. Carnaroli:Microwaving	***	0,0006
Carnaroli:Stewing vs. Vialone nano:Raw	****	<0,0001
Carnaroli:Stewing vs. Vialone nano:Boiling	****	<0,0001

Carnaroli:Stewing vs. Vialone nano:Stewing	****	<0,0001
Carnaroli:Stewing vs. Vialone nano:Microwaving	***	0,0008
Carnaroli:Stewing vs. Fragrance:Raw	****	<0,0001
Carnaroli:Stewing vs. Fragrance:Boiling	****	<0,0001
Carnaroli:Stewing vs. Fragrance:Stewing	****	<0,0001
Carnaroli:Stewing vs. Fragrance:Microwaving	ns	0,9526
Carnaroli:Stewing vs. Arborio:Raw	****	<0,0001
Carnaroli:Stewing vs. Arborio:Boiling	****	<0,0001
Carnaroli:Stewing vs. Arborio:Stewing	****	<0,0001
Carnaroli:Stewing vs. Arborio:Microwaving	**	0,0055
Carnaroli:Microwaving vs. Vialone nano:Raw	****	<0,0001
Carnaroli:Microwaving vs. Vialone nano:Boiling	****	<0,0001
Carnaroli:Microwaving vs. Vialone nano:Stewing	****	<0,0001
Carnaroli:Microwaving vs. Vialone nano:Microwaving	****	<0,0001
Carnaroli:Microwaving vs. Fragrance:Raw	****	<0,0001
Carnaroli:Microwaving vs. Fragrance:Boiling	****	<0,0001
Carnaroli:Microwaving vs. Fragrance:Stewing	****	<0,0001
Carnaroli:Microwaving vs. Fragrance:Microwaving	ns	0,1497
Carnaroli:Microwaving vs. Arborio:Raw	****	<0,0001
Carnaroli:Microwaving vs. Arborio:Boiling	****	<0,0001
Carnaroli:Microwaving vs. Arborio:Stewing	****	<0,0001
Carnaroli:Microwaving vs. Arborio:Microwaving	ns	>0,9999
Vialone nano:Raw vs. Vialone nano:Boiling	****	<0,0001
Vialone nano:Raw vs. Vialone nano:Stewing	****	<0,0001
Vialone nano:Raw vs. Vialone nano:Microwaving	****	<0,0001
Vialone nano:Raw vs. Fragrance:Raw	ns	0,9700
Vialone nano:Raw vs. Fragrance:Boiling	****	<0,0001
Vialone nano:Raw vs. Fragrance:Stewing	****	<0,0001
Vialone nano:Raw vs. Fragrance:Microwaving	****	<0,0001
Vialone nano:Raw vs. Arborio:Raw	ns	>0,9999
Vialone nano:Raw vs. Arborio:Boiling	****	<0,0001
Vialone nano:Raw vs. Arborio:Stewing	****	<0,0001
Vialone nano:Raw vs. Arborio:Microwaving	****	<0,0001
Vialone nano:Boiling vs. Vialone nano:Stewing	****	<0,0001
Vialone nano:Boiling vs. Vialone nano:Microwaving	****	<0,0001
Vialone nano:Boiling vs. Fragrance:Raw	****	<0,0001
Vialone nano:Boiling vs. Fragrance:Boiling	ns	>0,9999
Vialone nano:Boiling vs. Fragrance:Stewing	****	<0,0001
Vialone nano:Boiling vs. Fragrance:Microwaving	****	<0,0001
Vialone nano:Boiling vs. Arborio:Raw	****	<0,0001
Vialone nano:Boiling vs. Arborio:Boiling	****	<0,0001
Vialone nano:Boiling vs. Arborio:Stewing	****	<0,0001
Vialone nano:Boiling vs. Arborio:Microwaving	****	<0,0001
Vialone nano:Stewing vs. Vialone nano:Microwaving	****	<0,0001
Vialone nano:Stewing vs. Fragrance:Raw	****	<0,0001
Vialone nano:Stewing vs. Fragrance:Boiling	****	<0,0001
Vialone nano:Stewing vs. Fragrance:Stewing	****	<0,0001
Vialone nano:Stewing vs. Fragrance:Microwaving	****	<0,0001
Vialone nano:Stewing vs. Arborio:Raw	****	<0,0001
Vialone nano:Stewing vs. Arborio:Boiling	ns	0,9568
Vialone nano:Stewing vs. Arborio:Stewing	****	<0,0001
Vialone nano:Stewing vs. Arborio:Microwaving	****	<0,0001
Vialone nano:Microwaving vs. Fragrance:Raw	****	<0,0001
Vialone nano:Microwaving vs. Fragrance:Boiling	****	<0,0001
Vialone nano:Microwaving vs. Fragrance:Stewing	****	<0,0001
Vialone nano:Microwaving vs. Fragrance:Microwaving	****	<0,0001
Vialone nano:Microwaving vs. Arborio:Raw	****	<0,0001
Vialone nano:Microwaving vs. Arborio:Boiling	****	<0,0001
Vialone nano:Microwaving vs. Arborio:Stewing	****	<0,0001
Vialone nano:Microwaving vs. Arborio:Microwaving	****	<0,0001
Fragrance:Raw vs. Fragrance:Boiling	****	<0,0001
Fragrance:Raw vs. Fragrance:Stewing	****	<0,0001
Fragrance:Raw vs. Fragrance:Microwaving	****	<0,0001
Fragrance:Raw vs. Arborio:Raw	ns	0,9977

Fragrance:Raw vs. Arborio:Boiling	****	<0,0001
Fragrance:Raw vs. Arborio:Stewing	****	<0,0001
Fragrance:Raw vs. Arborio:Microwaving	****	<0,0001
Fragrance:Boiling vs. Fragrance:Stewing	****	<0,0001
Fragrance:Boiling vs. Fragrance:Microwaving	****	<0,0001
Fragrance:Boiling vs. Arborio:Raw	****	<0,0001
Fragrance:Boiling vs. Arborio:Boiling	****	<0,0001
Fragrance:Boiling vs. Arborio:Stewing	****	<0,0001
Fragrance:Boiling vs. Arborio:Microwaving	****	<0,0001
Fragrance:Stewing vs. Fragrance:Microwaving	****	<0,0001
Fragrance:Stewing vs. Arborio:Raw	****	<0,0001
Fragrance:Stewing vs. Arborio:Boiling	****	<0,0001
Fragrance:Stewing vs. Arborio:Stewing	****	<0,0001
Fragrance:Stewing vs. Arborio:Microwaving	****	<0,0001
Fragrance:Microwaving vs. Arborio:Raw	****	<0,0001
Fragrance:Microwaving vs. Arborio:Boiling	****	<0,0001
Fragrance:Microwaving vs. Arborio:Stewing	****	<0,0001
Fragrance:Microwaving vs. Arborio:Microwaving	ns	0,5261
Arborio:Raw vs. Arborio:Boiling	****	<0,0001
Arborio:Raw vs. Arborio:Stewing	****	<0,0001
Arborio:Raw vs. Arborio:Microwaving	****	<0,0001
Arborio:Boiling vs. Arborio:Stewing	****	<0,0001
Arborio:Boiling vs. Arborio:Microwaving	****	<0,0001
Arborio:Stewing vs. Arborio:Microwaving	****	<0,0001
