# Supplementary material for: Nutritional Properties of Rice Varieties Commonly Consumed in Italy and Applicability in Gluten Free Diet
Source: Foods. 2021 Jun 14;10(6):1375. doi: 10.3390/foods10061375 (PMC8232128; doi:10.3390/foods10061375)
Supplement: Supplementary file 1 [file foods-10-01375-s001.zip › Supplementary Materials 3 Flora.pdf]

Table S3. Tukey's multiple comparisons test of detectable amylose content.

Tukey's multiple comparisons test	Summary	Adjusted P Value
Ribe:Raw vs. Ribe:Boiling	****	<0,0001
Ribe:Raw vs. Ribe:Stewing	****	<0,0001
Ribe:Raw vs. Ribe:Microwaving	****	<0,0001
Ribe:Raw vs. Basmati:Raw	****	<0,0001
Ribe:Raw vs. Basmati:Boiling	****	<0,0001
Ribe:Raw vs. Basmati:Stewing	****	<0,0001
Ribe:Raw vs. Basmati:Microwaving	*	0,0115
Ribe:Raw vs. Carnaroli:Raw	****	<0,0001
Ribe:Raw vs. Carnaroli:Boiling	****	<0,0001
Ribe:Raw vs. Carnaroli:Stewing	****	<0,0001
Ribe:Raw vs. Carnaroli:Microwaving	****	<0,0001
Ribe:Raw vs. Vialone nano:Raw	****	<0,0001
Ribe:Raw vs. Vialone nano:Boiling	****	<0,0001
Ribe:Raw vs. Vialone nano:Stewing	****	<0,0001
Ribe:Raw vs. Vialone nano:Microwaving	****	<0,0001
Ribe:Raw vs. Fragrance:Raw	**	0,0043
Ribe:Raw vs. Fragrance:Boiling	****	<0,0001
Ribe:Raw vs. Fragrance:Stewing	****	<0,0001
Ribe:Raw vs. Fragrance:Microwaving	****	<0,0001
Ribe:Raw vs. Arborio:Raw	****	<0,0001
Ribe:Raw vs. Arborio:Boiling	****	<0,0001
Ribe:Raw vs. Arborio:Stewing	****	<0,0001
Ribe:Raw vs. Arborio:Microwaving	****	<0,0001
Ribe:Boiling vs. Ribe:Stewing	****	<0,0001
Ribe:Boiling vs. Ribe:Microwaving	****	<0,0001
Ribe:Boiling vs. Basmati:Raw	****	<0,0001
Ribe:Boiling vs. Basmati:Boiling	****	<0,0001
Ribe:Boiling vs. Basmati:Stewing	****	<0,0001
Ribe:Boiling vs. Basmati:Microwaving	****	<0,0001
Ribe:Boiling vs. Carnaroli:Raw	****	<0,0001
Ribe:Boiling vs. Carnaroli:Boiling	****	<0,0001
Ribe:Boiling vs. Carnaroli:Stewing	****	<0,0001
Ribe:Boiling vs. Carnaroli:Microwaving	****	<0,0001
Ribe:Boiling vs. Vialone nano:Raw	****	<0,0001
Ribe:Boiling vs. Vialone nano:Boiling	ns	0,1791
Ribe:Boiling vs. Vialone nano:Stewing	****	<0,0001
Ribe:Boiling vs. Vialone nano:Microwaving	****	<0,0001
Ribe:Boiling vs. Fragrance:Raw	****	<0,0001
Ribe:Boiling vs. Fragrance:Boiling	****	<0,0001
Ribe:Boiling vs. Fragrance:Stewing	****	<0,0001
Ribe:Boiling vs. Fragrance:Microwaving	****	<0,0001
Ribe:Boiling vs. Arborio:Raw	****	<0,0001
Ribe:Boiling vs. Arborio:Boiling	****	<0,0001
Ribe:Boiling vs. Arborio:Stewing	****	<0,0001
Ribe:Boiling vs. Arborio:Microwaving	****	<0,0001
Ribe:Stewing vs. Ribe:Microwaving	****	<0,0001
Ribe:Stewing vs. Basmati:Raw	****	<0,0001
Ribe:Stewing vs. Basmati:Boiling	***	0,0003
Ribe:Stewing vs. Basmati:Stewing	****	<0,0001
Ribe:Stewing vs. Basmati:Microwaving	****	<0,0001
Ribe:Stewing vs. Carnaroli:Raw	****	<0,0001
Ribe:Stewing vs. Carnaroli:Boiling	****	<0,0001
Ribe:Stewing vs. Carnaroli:Stewing	**	0,0011
Ribe:Stewing vs. Carnaroli:Microwaving	****	<0,0001
Ribe:Stewing vs. Vialone nano:Raw	****	<0,0001
Ribe:Stewing vs. Vialone nano:Boiling	****	<0,0001
Ribe:Stewing vs. Vialone nano:Stewing	ns	0,0599
Ribe:Stewing vs. Vialone nano:Microwaving	****	<0,0001
Ribe:Stewing vs. Fragrance:Raw	****	<0,0001
Ribe:Stewing vs. Fragrance:Boiling	****	<0,0001
Ribe:Stewing vs. Fragrance:Stewing	****	<0,0001

Ribe:Stewing vs. Fragrance:Microwaving	****	<0,0001
Ribe:Stewing vs. Arborio:Raw	****	<0,0001
Ribe:Stewing vs. Arborio:Boiling	****	<0,0001
Ribe:Stewing vs. Arborio:Stewing	****	<0,0001
Ribe:Stewing vs. Arborio:Microwaving	****	<0,0001
Ribe:Microwaving vs. Basmati:Raw	****	<0,0001
Ribe:Microwaving vs. Basmati:Boiling	****	<0,0001
Ribe:Microwaving vs. Basmati:Stewing	ns	0,1817
Ribe:Microwaving vs. Basmati:Microwaving	****	<0,0001
Ribe:Microwaving vs. Carnaroli:Raw	****	<0,0001
Ribe:Microwaving vs. Carnaroli:Boiling	****	<0,0001
Ribe:Microwaving vs. Carnaroli:Stewing	****	<0,0001
Ribe:Microwaving vs. Carnaroli:Microwaving	**	0,0016
Ribe:Microwaving vs. Vialone nano:Raw	****	<0,0001
Ribe:Microwaving vs. Vialone nano:Boiling	****	<0,0001
Ribe:Microwaving vs. Vialone nano:Stewing	****	<0,0001
Ribe:Microwaving vs. Vialone nano:Microwaving	**	0,0059
Ribe:Microwaving vs. Fragrance:Raw	****	<0,0001
Ribe:Microwaving vs. Fragrance:Boiling	****	<0,0001
Ribe:Microwaving vs. Fragrance:Stewing	*	0,0116
Ribe:Microwaving vs. Fragrance:Microwaving	****	<0,0001
Ribe:Microwaving vs. Arborio:Raw	****	<0,0001
Ribe:Microwaving vs. Arborio:Boiling	****	<0,0001
Ribe:Microwaving vs. Arborio:Stewing	*	0,0410
Ribe:Microwaving vs. Arborio:Microwaving	****	<0,0001
Basmati:Raw vs. Basmati:Boiling	****	<0,0001
Basmati:Raw vs. Basmati:Stewing	****	<0,0001
Basmati:Raw vs. Basmati:Microwaving	****	<0,0001
Basmati:Raw vs. Carnaroli:Raw	****	<0,0001
Basmati:Raw vs. Carnaroli:Boiling	****	<0,0001
Basmati:Raw vs. Carnaroli:Stewing	****	<0,0001
Basmati:Raw vs. Carnaroli:Microwaving	****	<0,0001
Basmati:Raw vs. Vialone nano:Raw	****	<0,0001
Basmati:Raw vs. Vialone nano:Boiling	****	<0,0001
Basmati:Raw vs. Vialone nano:Stewing	****	<0,0001
Basmati:Raw vs. Vialone nano:Microwaving	****	<0,0001
Basmati:Raw vs. Fragrance:Raw	****	<0,0001
Basmati:Raw vs. Fragrance:Boiling	****	<0,0001
Basmati:Raw vs. Fragrance:Stewing	****	<0,0001
Basmati:Raw vs. Fragrance:Microwaving	****	<0,0001
Basmati:Raw vs. Arborio:Raw	****	<0,0001
Basmati:Raw vs. Arborio:Boiling	****	<0,0001
Basmati:Raw vs. Arborio:Stewing	****	<0,0001
Basmati:Raw vs. Arborio:Microwaving	****	<0,0001
Basmati:Boiling vs. Basmati:Stewing	****	<0,0001
Basmati:Boiling vs. Basmati:Microwaving	****	<0,0001
Basmati:Boiling vs. Carnaroli:Raw	****	<0,0001
Basmati:Boiling vs. Carnaroli:Boiling	****	<0,0001
Basmati:Boiling vs. Carnaroli:Stewing	****	<0,0001
Basmati:Boiling vs. Carnaroli:Microwaving	****	<0,0001
Basmati:Boiling vs. Vialone nano:Raw	****	<0,0001
Basmati:Boiling vs. Vialone nano:Boiling	****	<0,0001
Basmati:Boiling vs. Vialone nano:Stewing	****	<0,0001
Basmati:Boiling vs. Vialone nano:Microwaving	****	<0,0001
Basmati:Boiling vs. Fragrance:Raw	****	<0,0001
Basmati:Boiling vs. Fragrance:Boiling	****	<0,0001
Basmati:Boiling vs. Fragrance:Stewing	****	<0,0001
Basmati:Boiling vs. Fragrance:Microwaving	****	<0,0001
Basmati:Boiling vs. Arborio:Raw	****	<0,0001
Basmati:Boiling vs. Arborio:Boiling	****	<0,0001
Basmati:Boiling vs. Arborio:Stewing	****	<0,0001
Basmati:Boiling vs. Arborio:Microwaving	****	<0,0001
Basmati:Stewing vs. Basmati:Microwaving	****	<0,0001
Basmati:Stewing vs. Carnaroli:Raw	****	<0,0001

Basmati:Stewing vs. Carnaroli:Boiling	****	<0,0001
Basmati:Stewing vs. Carnaroli:Stewing	****	<0,0001
Basmati:Stewing vs. Carnaroli:Microwaving	****	<0,0001
Basmati:Stewing vs. Vialone nano:Raw	****	<0,0001
Basmati:Stewing vs. Vialone nano:Boiling	****	<0,0001
Basmati:Stewing vs. Vialone nano:Stewing	****	<0,0001
Basmati:Stewing vs. Vialone nano:Microwaving	****	<0,0001
Basmati:Stewing vs. Fragrance:Raw	****	<0,0001
Basmati:Stewing vs. Fragrance:Boiling	****	<0,0001
Basmati:Stewing vs. Fragrance:Stewing	ns	>0,9999
Basmati:Stewing vs. Fragrance:Microwaving	****	<0,0001
Basmati:Stewing vs. Arborio:Raw	****	<0,0001
Basmati:Stewing vs. Arborio:Boiling	****	<0,0001
Basmati:Stewing vs. Arborio:Stewing	ns	>0,9999
Basmati:Stewing vs. Arborio:Microwaving	****	<0,0001
Basmati:Microwaving vs. Carnaroli:Raw	****	<0,0001
Basmati:Microwaving vs. Carnaroli:Boiling	****	<0,0001
Basmati:Microwaving vs. Carnaroli:Stewing	****	<0,0001
Basmati:Microwaving vs. Carnaroli:Microwaving	****	<0,0001
Basmati:Microwaving vs. Vialone nano:Raw	****	<0,0001
Basmati:Microwaving vs. Vialone nano:Boiling	****	<0,0001
Basmati:Microwaving vs. Vialone nano:Stewing	****	<0,0001
Basmati:Microwaving vs. Vialone nano:Microwaving	****	<0,0001
Basmati:Microwaving vs. Fragrance:Raw	****	<0,0001
Basmati:Microwaving vs. Fragrance:Boiling	****	<0,0001
Basmati:Microwaving vs. Fragrance:Stewing	****	<0,0001
Basmati:Microwaving vs. Fragrance:Microwaving	ns	0,9955
Basmati:Microwaving vs. Arborio:Raw	****	<0,0001
Basmati:Microwaving vs. Arborio:Boiling	****	<0,0001
Basmati:Microwaving vs. Arborio:Stewing	****	<0,0001
Basmati:Microwaving vs. Arborio:Microwaving	ns	0,0681
Carnaroli:Raw vs. Carnaroli:Boiling	****	<0,0001
Carnaroli:Raw vs. Carnaroli:Stewing	****	<0,0001
Carnaroli:Raw vs. Carnaroli:Microwaving	****	<0,0001
Carnaroli:Raw vs. Vialone nano:Raw	ns	>0,9999
Carnaroli:Raw vs. Vialone nano:Boiling	****	<0,0001
Carnaroli:Raw vs. Vialone nano:Stewing	****	<0,0001
Carnaroli:Raw vs. Vialone nano:Microwaving	****	<0,0001
Carnaroli:Raw vs. Fragrance:Raw	****	<0,0001
Carnaroli:Raw vs. Fragrance:Boiling	****	<0,0001
Carnaroli:Raw vs. Fragrance:Stewing	***	0,0008
Carnaroli:Raw vs. Fragrance:Microwaving	****	<0,0001
Carnaroli:Raw vs. Arborio:Raw	****	<0,0001
Carnaroli:Raw vs. Arborio:Boiling	****	<0,0001
Carnaroli:Raw vs. Arborio:Stewing	***	0,0002
Carnaroli:Raw vs. Arborio:Microwaving	*	0,0166
Carnaroli:Boiling vs. Carnaroli:Stewing	****	<0,0001
Carnaroli:Boiling vs. Carnaroli:Microwaving	****	<0,0001
Carnaroli:Boiling vs. Vialone nano:Raw	****	<0,0001
Carnaroli:Boiling vs. Vialone nano:Boiling	****	<0,0001
Carnaroli:Boiling vs. Vialone nano:Stewing	****	<0,0001
Carnaroli:Boiling vs. Vialone nano:Microwaving	****	<0,0001
Carnaroli:Boiling vs. Fragrance:Raw	****	<0,0001
Carnaroli:Boiling vs. Fragrance:Boiling	****	<0,0001
Carnaroli:Boiling vs. Fragrance:Stewing	****	<0,0001
Carnaroli:Boiling vs. Fragrance:Microwaving	****	<0,0001
Carnaroli:Boiling vs. Arborio:Raw	****	<0,0001
Carnaroli:Boiling vs. Arborio:Boiling	****	<0,0001
Carnaroli:Boiling vs. Arborio:Stewing	****	<0,0001
Carnaroli:Boiling vs. Arborio:Microwaving	****	<0,0001
Carnaroli:Stewing vs. Carnaroli:Microwaving	****	<0,0001
Carnaroli:Stewing vs. Vialone nano:Raw	****	<0,0001
Carnaroli:Stewing vs. Vialone nano:Boiling	****	<0,0001
Carnaroli:Stewing vs. Vialone nano:Stewing	ns	0,9993

Carnaroli:Stewing vs. Vialone nano:Microwaving	****	<0,0001
Carnaroli:Stewing vs. Fragrance:Raw	****	<0,0001
Carnaroli:Stewing vs. Fragrance:Boiling	****	<0,0001
Carnaroli:Stewing vs. Fragrance:Stewing	****	<0,0001
Carnaroli:Stewing vs. Fragrance:Microwaving	****	<0,0001
Carnaroli:Stewing vs. Arborio:Raw	****	<0,0001
Carnaroli:Stewing vs. Arborio:Boiling	****	<0,0001
Carnaroli:Stewing vs. Arborio:Stewing	****	<0,0001
Carnaroli:Stewing vs. Arborio:Microwaving	****	<0,0001
Carnaroli:Microwaving vs. Vialone nano:Raw	****	<0,0001
Carnaroli:Microwaving vs. Vialone nano:Boiling	****	<0,0001
Carnaroli:Microwaving vs. Vialone nano:Stewing	****	<0,0001
Carnaroli:Microwaving vs. Vialone nano:Microwaving	ns	>0,9999
Carnaroli:Microwaving vs. Fragrance:Raw	****	<0,0001
Carnaroli:Microwaving vs. Fragrance:Boiling	****	<0,0001
Carnaroli:Microwaving vs. Fragrance:Stewing	****	<0,0001
Carnaroli:Microwaving vs. Fragrance:Microwaving	****	<0,0001
Carnaroli:Microwaving vs. Arborio:Raw	****	<0,0001
Carnaroli:Microwaving vs. Arborio:Boiling	****	<0,0001
Carnaroli:Microwaving vs. Arborio:Stewing	****	<0,0001
Carnaroli:Microwaving vs. Arborio:Microwaving	****	<0,0001
Vialone nano:Raw vs. Vialone nano:Boiling	****	<0,0001
Vialone nano:Raw vs. Vialone nano:Stewing	****	<0,0001
Vialone nano:Raw vs. Vialone nano:Microwaving	****	<0,0001
Vialone nano:Raw vs. Fragrance:Raw	****	<0,0001
Vialone nano:Raw vs. Fragrance:Boiling	****	<0,0001
Vialone nano:Raw vs. Fragrance:Stewing	**	0,0019
Vialone nano:Raw vs. Fragrance:Microwaving	****	<0,0001
Vialone nano:Raw vs. Arborio:Raw	****	<0,0001
Vialone nano:Raw vs. Arborio:Boiling	****	<0,0001
Vialone nano:Raw vs. Arborio:Stewing	***	0,0004
Vialone nano:Raw vs. Arborio:Microwaving	**	0,0075
Vialone nano:Boiling vs. Vialone nano:Stewing	****	<0,0001
Vialone nano:Boiling vs. Vialone nano:Microwaving	****	<0,0001
Vialone nano:Boiling vs. Fragrance:Raw	****	<0,0001
Vialone nano:Boiling vs. Fragrance:Boiling	**	0,0040
Vialone nano:Boiling vs. Fragrance:Stewing	****	<0,0001
Vialone nano:Boiling vs. Fragrance:Microwaving	****	<0,0001
Vialone nano:Boiling vs. Arborio:Raw	****	<0,0001
Vialone nano:Boiling vs. Arborio:Boiling	****	<0,0001
Vialone nano:Boiling vs. Arborio:Stewing	****	<0,0001
Vialone nano:Boiling vs. Arborio:Microwaving	****	<0,0001
Vialone nano:Stewing vs. Vialone nano:Microwaving	****	<0,0001
Vialone nano:Stewing vs. Fragrance:Raw	****	<0,0001
Vialone nano:Stewing vs. Fragrance:Boiling	****	<0,0001
Vialone nano:Stewing vs. Fragrance:Stewing	****	<0,0001
Vialone nano:Stewing vs. Fragrance:Microwaving	****	<0,0001
Vialone nano:Stewing vs. Arborio:Raw	****	<0,0001
Vialone nano:Stewing vs. Arborio:Boiling	****	<0,0001
Vialone nano:Stewing vs. Arborio:Stewing	****	<0,0001
Vialone nano:Stewing vs. Arborio:Microwaving	****	<0,0001
Vialone nano:Microwaving vs. Fragrance:Raw	****	<0,0001
Vialone nano:Microwaving vs. Fragrance:Boiling	****	<0,0001
Vialone nano:Microwaving vs. Fragrance:Stewing	****	<0,0001
Vialone nano:Microwaving vs. Fragrance:Microwaving	****	<0,0001
Vialone nano:Microwaving vs. Arborio:Raw	****	<0,0001
Vialone nano:Microwaving vs. Arborio:Boiling	****	<0,0001
Vialone nano:Microwaving vs. Arborio:Stewing	****	<0,0001
Vialone nano:Microwaving vs. Arborio:Microwaving	****	<0,0001
Fragrance:Raw vs. Fragrance:Boiling	****	<0,0001
Fragrance:Raw vs. Fragrance:Stewing	****	<0,0001
Fragrance:Raw vs. Fragrance:Microwaving	****	<0,0001
Fragrance:Raw vs. Arborio:Raw	ns	0,9993
Fragrance:Raw vs. Arborio:Boiling	****	<0,0001

Fragrance:Raw vs. Arborio:Stewing	****	<0,0001
Fragrance:Raw vs. Arborio:Microwaving	****	<0,0001
Fragrance:Boiling vs. Fragrance:Stewing	****	<0,0001
Fragrance:Boiling vs. Fragrance:Microwaving	****	<0,0001
Fragrance:Boiling vs. Arborio:Raw	****	<0,0001
Fragrance:Boiling vs. Arborio:Boiling	*	0,0496
Fragrance:Boiling vs. Arborio:Stewing	****	<0,0001
Fragrance:Boiling vs. Arborio:Microwaving	****	<0,0001
Fragrance:Stewing vs. Fragrance:Microwaving	****	<0,0001
Fragrance:Stewing vs. Arborio:Raw	****	<0,0001
Fragrance:Stewing vs. Arborio:Boiling	****	<0,0001
Fragrance:Stewing vs. Arborio:Stewing	ns	>0,9999
Fragrance:Stewing vs. Arborio:Microwaving	****	<0,0001
Fragrance:Microwaving vs. Arborio:Raw	****	<0,0001
Fragrance:Microwaving vs. Arborio:Boiling	****	<0,0001
Fragrance:Microwaving vs. Arborio:Stewing	****	<0,0001
Fragrance:Microwaving vs. Arborio:Microwaving	ns	0,8595
Arborio:Raw vs. Arborio:Boiling	****	<0,0001
Arborio:Raw vs. Arborio:Stewing	****	<0,0001
Arborio:Raw vs. Arborio:Microwaving	****	<0,0001
Arborio:Boiling vs. Arborio:Stewing	****	<0,0001
Arborio:Boiling vs. Arborio:Microwaving	****	<0,0001
Arborio:Stewing vs. Arborio:Microwaving	****	<0,0001
