# Supplementary material for: Nutritional Properties of Rice Varieties Commonly Consumed in Italy and Applicability in Gluten Free Diet
Source: Foods. 2021 Jun 14;10(6):1375. doi: 10.3390/foods10061375 (PMC8232128; doi:10.3390/foods10061375)
Supplement: Supplementary file 1 [file foods-10-01375-s001.zip › Supplementary Materials 4 Flora.pdf]

Table S4. Tukey's multiple comparisons test of detectable amylose/total CHO content after cooking processes.

Tukey's multiple comparisons test	Summary	Adjusted P Value
Ribe:Raw vs. Ribe:Boiling	ns	0,9997
Ribe:Raw vs. Ribe:Stewing	ns	0,2234
Ribe:Raw vs. Ribe:Microwaving	*	0,0400
Ribe:Raw vs. Basmati:Raw	****	<0,0001
Ribe:Raw vs. Basmati:Boiling	****	<0,0001
Ribe:Raw vs. Basmati:Stewing	****	<0,0001
Ribe:Raw vs. Basmati:Microwaving	ns	0,1249
Ribe:Raw vs. Carnaroli:Raw	**	0,0012
Ribe:Raw vs. Carnaroli:Boiling	****	<0,0001
Ribe:Raw vs. Carnaroli:Stewing	****	<0,0001
Ribe:Raw vs. Carnaroli:Microwaving	****	<0,0001
Ribe:Raw vs. Vialone nano:Raw	****	<0,0001
Ribe:Raw vs. Vialone nano:Boiling	ns	0,9261
Ribe:Raw vs. Vialone nano:Stewing	****	<0,0001
Ribe:Raw vs. Vialone nano:Microwaving	ns	0,8414
Ribe:Raw vs. Fragrance:Raw	ns	0,7547
Ribe:Raw vs. Fragrance:Boiling	ns	0,1372
Ribe:Raw vs. Fragrance:Stewing	****	<0,0001
Ribe:Raw vs. Fragrance:Microwaving	**	0,0100
Ribe:Raw vs. Arborio:Raw	ns	0,8419
Ribe:Raw vs. Arborio:Boiling	****	<0,0001
Ribe:Raw vs. Arborio:Stewing	****	<0,0001
Ribe:Raw vs. Arborio:Microwaving	ns	0,9832
Ribe:Boiling vs. Ribe:Stewing	**	0,0085
Ribe:Boiling vs. Ribe:Microwaving	ns	0,5502
Ribe:Boiling vs. Basmati:Raw	****	<0,0001
Ribe:Boiling vs. Basmati:Boiling	****	<0,0001
Ribe:Boiling vs. Basmati:Stewing	****	<0,0001
Ribe:Boiling vs. Basmati:Microwaving	**	0,0037
Ribe:Boiling vs. Carnaroli:Raw	ns	0,0512
Ribe:Boiling vs. Carnaroli:Boiling	****	<0,0001
Ribe:Boiling vs. Carnaroli:Stewing	****	<0,0001
Ribe:Boiling vs. Carnaroli:Microwaving	****	<0,0001
Ribe:Boiling vs. Vialone nano:Raw	***	0,0008
Ribe:Boiling vs. Vialone nano:Boiling	ns	0,1955
Ribe:Boiling vs. Vialone nano:Stewing	****	<0,0001
Ribe:Boiling vs. Vialone nano:Microwaving	ns	>0,9999
Ribe:Boiling vs. Fragrance:Raw	ns	0,0867
Ribe:Boiling vs. Fragrance:Boiling	ns	0,8620
Ribe:Boiling vs. Fragrance:Stewing	****	<0,0001
Ribe:Boiling vs. Fragrance:Microwaving	***	0,0002
Ribe:Boiling vs. Arborio:Raw	ns	0,1249
Ribe:Boiling vs. Arborio:Boiling	****	<0,0001
Ribe:Boiling vs. Arborio:Stewing	****	<0,0001
Ribe:Boiling vs. Arborio:Microwaving	ns	0,3350
Ribe:Stewing vs. Ribe:Microwaving	****	<0,0001
Ribe:Stewing vs. Basmati:Raw	****	<0,0001
Ribe:Stewing vs. Basmati:Boiling	**	0,0017
Ribe:Stewing vs. Basmati:Stewing	****	<0,0001
Ribe:Stewing vs. Basmati:Microwaving	ns	>0,9999
Ribe:Stewing vs. Carnaroli:Raw	****	<0,0001
Ribe:Stewing vs. Carnaroli:Boiling	****	<0,0001
Ribe:Stewing vs. Carnaroli:Stewing	****	<0,0001
Ribe:Stewing vs. Carnaroli:Microwaving	****	<0,0001
Ribe:Stewing vs. Vialone nano:Raw	****	<0,0001
Ribe:Stewing vs. Vialone nano:Boiling	ns	0,9999
Ribe:Stewing vs. Vialone nano:Stewing	****	<0,0001
Ribe:Stewing vs. Vialone nano:Microwaving	***	0,0004
Ribe:Stewing vs. Fragrance:Raw	ns	>0,9999
Ribe:Stewing vs. Fragrance:Boiling	****	<0,0001
Ribe:Stewing vs. Fragrance:Stewing	****	<0,0001

Ribe:Stewing vs. Fragrance:Microwaving	ns	0,9999
Ribe:Stewing vs. Arborio:Raw	ns	>0,9999
Ribe:Stewing vs. Arborio:Boiling	****	<0,0001
Ribe:Stewing vs. Arborio:Stewing	****	<0,0001
Ribe:Stewing vs. Arborio:Microwaving	ns	0,9975
Ribe:Microwaving vs. Basmati:Raw	****	<0,0001
Ribe:Microwaving vs. Basmati:Boiling	****	<0,0001
Ribe:Microwaving vs. Basmati:Stewing	****	<0,0001
Ribe:Microwaving vs. Basmati:Microwaving	****	<0,0001
Ribe:Microwaving vs. Carnaroli:Raw	ns	>0,9999
Ribe:Microwaving vs. Carnaroli:Boiling	****	<0,0001
Ribe:Microwaving vs. Carnaroli:Stewing	****	<0,0001
Ribe:Microwaving vs. Carnaroli:Microwaving	*	0,0241
Ribe:Microwaving vs. Vialone nano:Raw	ns	0,6324
Ribe:Microwaving vs. Vialone nano:Boiling	****	<0,0001
Ribe:Microwaving vs. Vialone nano:Stewing	****	<0,0001
Ribe:Microwaving vs. Vialone nano:Microwaving	ns	0,9847
Ribe:Microwaving vs. Fragrance:Raw	****	<0,0001
Ribe:Microwaving vs. Fragrance:Boiling	ns	>0,9999
Ribe:Microwaving vs. Fragrance:Stewing	****	<0,0001
Ribe:Microwaving vs. Fragrance:Microwaving	****	<0,0001
Ribe:Microwaving vs. Arborio:Raw	****	<0,0001
Ribe:Microwaving vs. Arborio:Boiling	****	<0,0001
Ribe:Microwaving vs. Arborio:Stewing	****	<0,0001
Ribe:Microwaving vs. Arborio:Microwaving	***	0,0002
Basmati:Raw vs. Basmati:Boiling	****	<0,0001
Basmati:Raw vs. Basmati:Stewing	***	0,0010
Basmati:Raw vs. Basmati:Microwaving	****	<0,0001
Basmati:Raw vs. Carnaroli:Raw	****	<0,0001
Basmati:Raw vs. Carnaroli:Boiling	**	0,0020
Basmati:Raw vs. Carnaroli:Stewing	****	<0,0001
Basmati:Raw vs. Carnaroli:Microwaving	****	<0,0001
Basmati:Raw vs. Vialone nano:Raw	****	<0,0001
Basmati:Raw vs. Vialone nano:Boiling	****	<0,0001
Basmati:Raw vs. Vialone nano:Stewing	ns	0,5362
Basmati:Raw vs. Vialone nano:Microwaving	****	<0,0001
Basmati:Raw vs. Fragrance:Raw	****	<0,0001
Basmati:Raw vs. Fragrance:Boiling	****	<0,0001
Basmati:Raw vs. Fragrance:Stewing	ns	0,9899
Basmati:Raw vs. Fragrance:Microwaving	****	<0,0001
Basmati:Raw vs. Arborio:Raw	****	<0,0001
Basmati:Raw vs. Arborio:Boiling	****	<0,0001
Basmati:Raw vs. Arborio:Stewing	ns	0,0564
Basmati:Raw vs. Arborio:Microwaving	****	<0,0001
Basmati:Boiling vs. Basmati:Stewing	****	<0,0001
Basmati:Boiling vs. Basmati:Microwaving	**	0,0040
Basmati:Boiling vs. Carnaroli:Raw	****	<0,0001
Basmati:Boiling vs. Carnaroli:Boiling	ns	>0,9999
Basmati:Boiling vs. Carnaroli:Stewing	****	<0,0001
Basmati:Boiling vs. Carnaroli:Microwaving	****	<0,0001
Basmati:Boiling vs. Vialone nano:Raw	****	<0,0001
Basmati:Boiling vs. Vialone nano:Boiling	****	<0,0001
Basmati:Boiling vs. Vialone nano:Stewing	ns	0,1457
Basmati:Boiling vs. Vialone nano:Microwaving	****	<0,0001
Basmati:Boiling vs. Fragrance:Raw	***	0,0001
Basmati:Boiling vs. Fragrance:Boiling	****	<0,0001
Basmati:Boiling vs. Fragrance:Stewing	**	0,0093
Basmati:Boiling vs. Fragrance:Microwaving	ns	0,0597
Basmati:Boiling vs. Arborio:Raw	****	<0,0001
Basmati:Boiling vs. Arborio:Boiling	****	<0,0001
Basmati:Boiling vs. Arborio:Stewing	****	<0,0001
Basmati:Boiling vs. Arborio:Microwaving	****	<0,0001
Basmati:Stewing vs. Basmati:Microwaving	****	<0,0001
Basmati:Stewing vs. Carnaroli:Raw	****	<0,0001

Basmati:Stewing vs. Carnaroli:Boiling	****	<0,0001
Basmati:Stewing vs. Carnaroli:Stewing	****	<0,0001
Basmati:Stewing vs. Carnaroli:Microwaving	****	<0,0001
Basmati:Stewing vs. Vialone nano:Raw	****	<0,0001
Basmati:Stewing vs. Vialone nano:Boiling	****	<0,0001
Basmati:Stewing vs. Vialone nano:Stewing	****	<0,0001
Basmati:Stewing vs. Vialone nano:Microwaving	****	<0,0001
Basmati:Stewing vs. Fragrance:Raw	****	<0,0001
Basmati:Stewing vs. Fragrance:Boiling	****	<0,0001
Basmati:Stewing vs. Fragrance:Stewing	****	<0,0001
Basmati:Stewing vs. Fragrance:Microwaving	****	<0,0001
Basmati:Stewing vs. Arborio:Raw	****	<0,0001
Basmati:Stewing vs. Arborio:Boiling	****	<0,0001
Basmati:Stewing vs. Arborio:Stewing	ns	0,9992
Basmati:Stewing vs. Arborio:Microwaving	****	<0,0001
Basmati:Microwaving vs. Carnaroli:Raw	****	<0,0001
Basmati:Microwaving vs. Carnaroli:Boiling	****	<0,0001
Basmati:Microwaving vs. Carnaroli:Stewing	****	<0,0001
Basmati:Microwaving vs. Carnaroli:Microwaving	****	<0,0001
Basmati:Microwaving vs. Vialone nano:Raw	****	<0,0001
Basmati:Microwaving vs. Vialone nano:Boiling	ns	0,9978
Basmati:Microwaving vs. Vialone nano:Stewing	****	<0,0001
Basmati:Microwaving vs. Vialone nano:Microwaving	***	0,0002
Basmati:Microwaving vs. Fragrance:Raw	ns	>0,9999
Basmati:Microwaving vs. Fragrance:Boiling	****	<0,0001
Basmati:Microwaving vs. Fragrance:Stewing	****	<0,0001
Basmati:Microwaving vs. Fragrance:Microwaving	ns	>0,9999
Basmati:Microwaving vs. Arborio:Raw	ns	0,9997
Basmati:Microwaving vs. Arborio:Boiling	****	<0,0001
Basmati:Microwaving vs. Arborio:Stewing	****	<0,0001
Basmati:Microwaving vs. Arborio:Microwaving	ns	0,9813
Carnaroli:Raw vs. Carnaroli:Boiling	****	<0,0001
Carnaroli:Raw vs. Carnaroli:Stewing	****	<0,0001
Carnaroli:Raw vs. Carnaroli:Microwaving	ns	0,3645
Carnaroli:Raw vs. Vialone nano:Raw	ns	0,9991
Carnaroli:Raw vs. Vialone nano:Boiling	****	<0,0001
Carnaroli:Raw vs. Vialone nano:Stewing	****	<0,0001
Carnaroli:Raw vs. Vialone nano:Microwaving	ns	0,4006
Carnaroli:Raw vs. Fragrance:Raw	****	<0,0001
Carnaroli:Raw vs. Fragrance:Boiling	ns	0,9891
Carnaroli:Raw vs. Fragrance:Stewing	****	<0,0001
Carnaroli:Raw vs. Fragrance:Microwaving	****	<0,0001
Carnaroli:Raw vs. Arborio:Raw	****	<0,0001
Carnaroli:Raw vs. Arborio:Boiling	****	<0,0001
Carnaroli:Raw vs. Arborio:Stewing	****	<0,0001
Carnaroli:Raw vs. Arborio:Microwaving	****	<0,0001
Carnaroli:Boiling vs. Carnaroli:Stewing	****	<0,0001
Carnaroli:Boiling vs. Carnaroli:Microwaving	****	<0,0001
Carnaroli:Boiling vs. Vialone nano:Raw	****	<0,0001
Carnaroli:Boiling vs. Vialone nano:Boiling	****	<0,0001
Carnaroli:Boiling vs. Vialone nano:Stewing	ns	0,8273
Carnaroli:Boiling vs. Vialone nano:Microwaving	****	<0,0001
Carnaroli:Boiling vs. Fragrance:Raw	****	<0,0001
Carnaroli:Boiling vs. Fragrance:Boiling	****	<0,0001
Carnaroli:Boiling vs. Fragrance:Stewing	ns	0,1951
Carnaroli:Boiling vs. Fragrance:Microwaving	**	0,0019
Carnaroli:Boiling vs. Arborio:Raw	****	<0,0001
Carnaroli:Boiling vs. Arborio:Boiling	****	<0,0001
Carnaroli:Boiling vs. Arborio:Stewing	****	<0,0001
Carnaroli:Boiling vs. Arborio:Microwaving	****	<0,0001
Carnaroli:Stewing vs. Carnaroli:Microwaving	****	<0,0001
Carnaroli:Stewing vs. Vialone nano:Raw	****	<0,0001
Carnaroli:Stewing vs. Vialone nano:Boiling	****	<0,0001
Carnaroli:Stewing vs. Vialone nano:Stewing	****	<0,0001

Carnaroli:Stewing vs. Vialone nano:Microwaving	****	<0,0001
Carnaroli:Stewing vs. Fragrance:Raw	****	<0,0001
Carnaroli:Stewing vs. Fragrance:Boiling	****	<0,0001
Carnaroli:Stewing vs. Fragrance:Stewing	****	<0,0001
Carnaroli:Stewing vs. Fragrance:Microwaving	****	<0,0001
Carnaroli:Stewing vs. Arborio:Raw	****	<0,0001
Carnaroli:Stewing vs. Arborio:Boiling	*	0,0377
Carnaroli:Stewing vs. Arborio:Stewing	****	<0,0001
Carnaroli:Stewing vs. Arborio:Microwaving	****	<0,0001
Carnaroli:Microwaving vs. Vialone nano:Raw	ns	0,9949
Carnaroli:Microwaving vs. Vialone nano:Boiling	****	<0,0001
Carnaroli:Microwaving vs. Vialone nano:Stewing	****	<0,0001
Carnaroli:Microwaving vs. Vialone nano:Microwaving	***	0,0001
Carnaroli:Microwaving vs. Fragrance:Raw	****	<0,0001
Carnaroli:Microwaving vs. Fragrance:Boiling	**	0,0056
Carnaroli:Microwaving vs. Fragrance:Stewing	****	<0,0001
Carnaroli:Microwaving vs. Fragrance:Microwaving	****	<0,0001
Carnaroli:Microwaving vs. Arborio:Raw	****	<0,0001
Carnaroli:Microwaving vs. Arborio:Boiling	***	0,0001
Carnaroli:Microwaving vs. Arborio:Stewing	****	<0,0001
Carnaroli:Microwaving vs. Arborio:Microwaving	****	<0,0001
Vialone nano:Raw vs. Vialone nano:Boiling	****	<0,0001
Vialone nano:Raw vs. Vialone nano:Stewing	****	<0,0001
Vialone nano:Raw vs. Vialone nano:Microwaving	*	0,0156
Vialone nano:Raw vs. Fragrance:Raw	****	<0,0001
Vialone nano:Raw vs. Fragrance:Boiling	ns	0,3058
Vialone nano:Raw vs. Fragrance:Stewing	****	<0,0001
Vialone nano:Raw vs. Fragrance:Microwaving	****	<0,0001
Vialone nano:Raw vs. Arborio:Raw	****	<0,0001
Vialone nano:Raw vs. Arborio:Boiling	****	<0,0001
Vialone nano:Raw vs. Arborio:Stewing	****	<0,0001
Vialone nano:Raw vs. Arborio:Microwaving	****	<0,0001
Vialone nano:Boiling vs. Vialone nano:Stewing	****	<0,0001
Vialone nano:Boiling vs. Vialone nano:Microwaving	*	0,0176
Vialone nano:Boiling vs. Fragrance:Raw	ns	>0,9999
Vialone nano:Boiling vs. Fragrance:Boiling	***	0,0004
Vialone nano:Boiling vs. Fragrance:Stewing	****	<0,0001
Vialone nano:Boiling vs. Fragrance:Microwaving	ns	0,7288
Vialone nano:Boiling vs. Arborio:Raw	ns	>0,9999
Vialone nano:Boiling vs. Arborio:Boiling	****	<0,0001
Vialone nano:Boiling vs. Arborio:Stewing	****	<0,0001
Vialone nano:Boiling vs. Arborio:Microwaving	ns	>0,9999
Vialone nano:Stewing vs. Vialone nano:Microwaving	****	<0,0001
Vialone nano:Stewing vs. Fragrance:Raw	****	<0,0001
Vialone nano:Stewing vs. Fragrance:Boiling	****	<0,0001
Vialone nano:Stewing vs. Fragrance:Stewing	ns	>0,9999
Vialone nano:Stewing vs. Fragrance:Microwaving	****	<0,0001
Vialone nano:Stewing vs. Arborio:Raw	****	<0,0001
Vialone nano:Stewing vs. Arborio:Boiling	****	<0,0001
Vialone nano:Stewing vs. Arborio:Stewing	****	<0,0001
Vialone nano:Stewing vs. Arborio:Microwaving	****	<0,0001
Vialone nano:Microwaving vs. Fragrance:Raw	**	0,0061
Vialone nano:Microwaving vs. Fragrance:Boiling	ns	0,9998
Vialone nano:Microwaving vs. Fragrance:Stewing	****	<0,0001
Vialone nano:Microwaving vs. Fragrance:Microwaving	****	<0,0001
Vialone nano:Microwaving vs. Arborio:Raw	**	0,0097
Vialone nano:Microwaving vs. Arborio:Boiling	****	<0,0001
Vialone nano:Microwaving vs. Arborio:Stewing	****	<0,0001
Vialone nano:Microwaving vs. Arborio:Microwaving	*	0,0386
Fragrance:Raw vs. Fragrance:Boiling	***	0,0001
Fragrance:Raw vs. Fragrance:Stewing	****	<0,0001
Fragrance:Raw vs. Fragrance:Microwaving	ns	0,9124
Fragrance:Raw vs. Arborio:Raw	ns	>0,9999
Fragrance:Raw vs. Arborio:Boiling	****	<0,0001

Fragrance:Raw vs. Arborio:Stewing	****	<0,0001
Fragrance:Raw vs. Arborio:Microwaving	ns	>0,9999
Fragrance:Boiling vs. Fragrance:Stewing	****	<0,0001
Fragrance:Boiling vs. Fragrance:Microwaving	****	<0,0001
Fragrance:Boiling vs. Arborio:Raw	***	0,0002
Fragrance:Boiling vs. Arborio:Boiling	****	<0,0001
Fragrance:Boiling vs. Arborio:Stewing	****	<0,0001
Fragrance:Boiling vs. Arborio:Microwaving	***	0,0009
Fragrance:Stewing vs. Fragrance:Microwaving	****	<0,0001
Fragrance:Stewing vs. Arborio:Raw	****	<0,0001
Fragrance:Stewing vs. Arborio:Boiling	****	<0,0001
Fragrance:Stewing vs. Arborio:Stewing	***	0,0004
Fragrance:Stewing vs. Arborio:Microwaving	****	<0,0001
Fragrance:Microwaving vs. Arborio:Raw	ns	0,8466
Fragrance:Microwaving vs. Arborio:Boiling	****	<0,0001
Fragrance:Microwaving vs. Arborio:Stewing	****	<0,0001
Fragrance:Microwaving vs. Arborio:Microwaving	ns	0,5355
Arborio:Raw vs. Arborio:Boiling	****	<0,0001
Arborio:Raw vs. Arborio:Stewing	****	<0,0001
Arborio:Raw vs. Arborio:Microwaving	ns	>0,9999
Arborio:Boiling vs. Arborio:Stewing	****	<0,0001
Arborio:Boiling vs. Arborio:Microwaving	****	<0,0001
Arborio:Stewing vs. Arborio:Microwaving	****	<0,0001
